# Enriching Pourbaix diagrams for improved understanding of competitive electrochemical phases

**DOI:** 10.1038/s41524-026-02232-5

**Published:** 2026-07-22

**Authors:** Diego Garay-Ruiz, Marisol Martín-González

**Affiliations:** Instituto de Micro y Nanotecnología, IMN-CNM, CSIC (CEI UAM+CSIC), Isaac Newton, 8, Tres Cantos, Madrid Spain

**Keywords:** Chemistry, Materials science, Mathematics and computing

## Abstract

Pourbaix diagrams are a widespread tool in electrochemistry, enabling a direct understanding of how phase stability changes with potential and pH. While originally founded on a combination of theoretical equations and experimental data, in recent years, there has been a growing interest in the *prediction* of this kind of diagram by means of computational data, fueled by the success of massive databases such as the Materials Project. Herein, we propose a novel approach based on the calculation of thermodynamic Boltzmann populations across the pH and potential space, constructing 3D *Pourbaix spaces*. From there, we introduce competition-informed Pourbaix diagrams, where the representation of the *prevalence* of the major phase reveals regions where multiple phases are nearly degenerate, thereby identifying conditions where phase assignments are ill-defined, as energy differences between phases fall inside the DFT standard error range—providing thermodynamic confidence in the assignment of dominant phases across the diagram. Furthermore, we propose additional analyses on the computed populations to improve the characterization of competitive phases in regions of interest (speciation diagrams) and to simplify the comparison of inter-system Pourbaix diagrams. We implement this framework in PourbaixUP, an open-source tool enabling effective and detailed analysis of binary, ternary and higher-order systems, which demonstrate through several examples: binary metal combinations, passivation and corrosion windows in Fe/Cr alloys and chalcogenide materials of interest in thermoelectricity.

## Introduction

One of the most fundamental questions tackled by Chemistry and Materials Science is knowing which species are stable under a given set of conditions, and how changes in these conditions affect this stability. This challenge permeates all subfields of chemistry, mainly altering which are the particular conditions that need to be considered. On the one hand, a direct way to answer this question is to set up controlled experiments and characterize the molecules or material phases that are obtained. On the other hand, computational studies have been gaining importance throughout the last decades, developing models that enable realistic *predictions* to complement the experiments and improve the overall understanding.

In the particular case of electrochemistry, the applied electric potential and the pH range of the target solution are two of the most relevant factors to take into account. Consequently, Pourbaix diagrams^1^, a kind of phase diagram representing the most thermodynamically stable phases of a given chemical system with varying pH and potential, are an invaluable tool for practical electrochemistry. Over the years, a sheer amount of Pourbaix diagrams have been collected and reported, determining thermodynamic stabilities for a wide variety of element combinations. While these diagrams have traditionally been built by combining experimental data with simple theoretical frameworks (e.g., Nernst equation)^2–4^, there is an increasing interest in their computational prediction, by means of ab initio or DFT calculations, to tackle problems in battery stability^5^ or electrocatalysis^6–9^. Recent efforts^10–12^ have exploited the success of massive databases for Computational Materials Science, such as the Materials Project^13^. This platform not only hosts an enormous amount of DFT-derived thermodynamic stability information, but also provides tools^10^ to introduce pH and potential effects and thus generate the corresponding Pourbaix diagrams. Computational approaches are of particular importance when tackling the huge combinatorial space that arises when considering binary, ternary or higher-order combinations of elements, for which experimental Pourbaix diagrams are not systematically available. It is important to note that, by definition, only *thermodynamic* effects are regarded in this kind of representation, without considering the kinetics of the process—e.g., if the formation of the stable phase is too slow, it might not be observed in the experimental setup.

The success of Pourbaix diagrams (and, more generally, of phase diagrams) comes from their simple interpretation—well-defined regions that clarify the pH and potential ranges in which a phase will be thermodynamically stable. However, this simplicity can lead to oversimplification: since only the lowest-energy phase is depicted, no consideration is given to how competitive other materials might be. As we move to explore materials and mixtures of increasing complexity, and also as we consider high-throughput calculations as our data source, this might become an important source of error. It is easy to picture a situation where two phases can be very close in energy, with differences being smaller than the DFT error in the range of 10–100 meV/atom^14,15^ associated with the GGA functionals such as PBE^16,17^, employed in the Materials Project. The phase (Pourbaix) diagram will only select one of the two competitors, not only plainly disregarding the other one, but also assuming this ill-defined region to be as reliable as other regions of the diagram where the assignment of the major phase is clearer.

Recent works have already tackled the uncertainty of Pourbaix diagrams, in the context of addressing the confidence in a given reaction mechanism by means of functional benchmarking^18^, diminishing the actual DFT error for different compound families^15^ or Machine Learning-based Bayesian error estimations for metallic nanoparticles^19^. On the other hand, other efforts have focused on diminishing the DFT-associated error by employing functionals that improve the prediction of aqueous stability upon PBE, such as SCAN^20^, or defining ad-hoc corrections for compound families in the context of the Materials Project^15^. Nonetheless, the focus there is on the uncertainty associated with the *energy* values used to construct the diagram, not on the intrinsic loss of information due to the dimensionality reduction performed when building the diagram. In this context, addressing this information loss by characterizing the actual *prevalence* of the involved phases would provide a direct assessment of which regions of the phase space are more susceptible to requiring further corrections.

In this communication, we face this challenge by introducing these considerations in the Pourbaix diagram by means of computing the *populations* of each phase at different (pH, potential) conditions. In this way, it becomes possible to determine how *prevalent* the assigned major phase is along the whole diagram, adding a new layer of information that is absent from traditional analyses. This procedure is integrated with the existing toolkit from the Materials Project and requires minimal computational overhead, thus strongly facilitating its integration with existing pipelines and consequently its adoption by the community. Furthermore, we also explore these effects in more detail with alternative representations and analyses, with the ultimate goal of providing a simple but robust framework to tackle complex electrochemical systems.

## Results and discussion

### Metal combinations

As a first demonstration of the capabilities of our approach, we considered a set of binary combinations of common transition metals: namely, Ag, Cu, Fe and Ni. For simplicity, we chose 50:50 A:B compositions for all six cases—nevertheless, any other arbitrary elemental ratio could be considered instead.

**Fig. 1:**
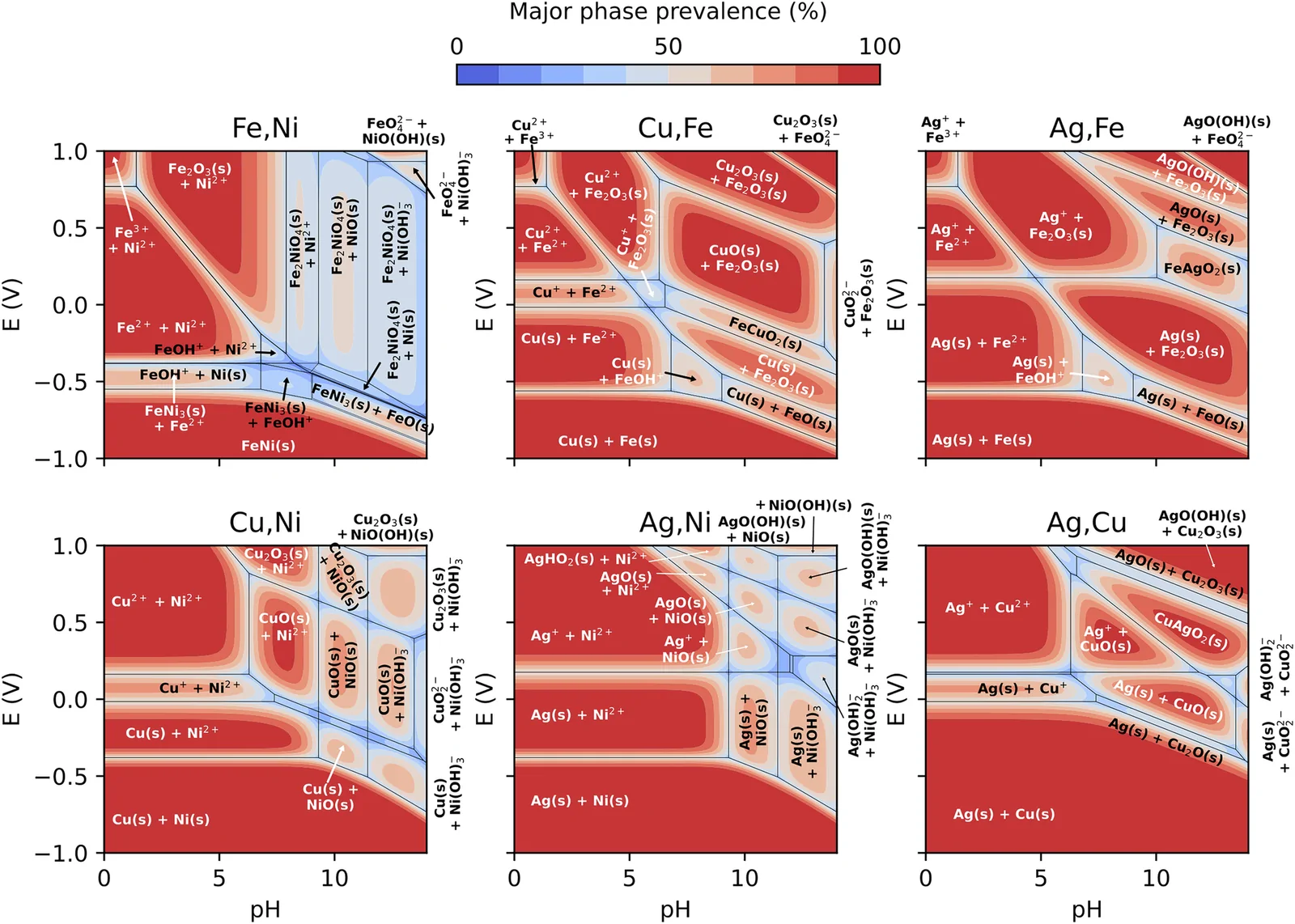
Boltzmann-weighted Pourbaix diagrams for A/B metal combinations. 50% A, 50% B, V range (vs. SHE) −1.0 V to 1.0 V, pH range 0 to 14. The color scale indicates the Boltzmann population (prevalence) of the major phase. Red/warm colors denote single dominant phases ( > 80%), blue/gray colors ( < 70%) denote regions where Boltzmann populations are distributed across multiple phases, indicating strong phase competition due to near-degenerate formation energies.

All of these correspond to extensively studied systems^21,22^, and thus provide a good starting point to showcase our extended Pourbaix diagrams. First of all, we represented the contour plot with the prevalence of the major phase for each of the six possible metal combinations, and overlaid the lines and labels defining the “traditional” Pourbaix diagram, as depicted in Fig. 1. The plotting approach is analogous to that of Sumaria and co-workers^18^: nevertheless, the scope is widely different—in our case, we are representing the intrinsic free-energy-derived phase prevalences computed for an individual system, while their approach considers DFT uncertainties characterized by a functional benchmarking and the consideration of different compound families.

The Boltzmann-weighted Pourbaix diagram in Fig. 1 displays phase populations in a color scale across the pH-potential space. Regions dominated by a single thermodynamically stable phase appear in deep red colors (populations >80%): therein, the major phase is clearly dominant, and the competitiveness of any other species in the system is limited. In these cases, the assignment of the traditional Pourbaix diagram is accurate. In contrast, regions where Boltzmann populations are distributed across multiple phases indicate strong phase competition due to near-degenerate formation energies, and appear in blue-gray colors, accounting for major phase prevalences below 70%. Here, traditional Pourbaix assignments are most uncertain and could be misleading, as other phases are likely to influence the behavior of the system. A clear feature of the maps in Fig. 1 alloys is how phase transitions are naturally reflected in the prevalence values, as the lines of the original diagram coincide with low-prevalence regions. This is fully consistent with a realistic vision of how phase transitions are expected to occur: for an *A* → *B* transition to happen, a continuous decrease of the amount of A and a continuous increase of the amount of B will take place. Traditional diagrams, instead, depict harder (1-to-0) transitions where a sudden, unphysical switch from A to B is predicted. In consequence, this natural reflection of harsh Pourbaix limits in smoother prevalence-based limits arises as a remarkable validation of our approach and our core hypothesis: the existence of variations in the competitiveness between phases even within the limits of a single region in the Pourbaix diagram.

Looking at the different situations, we have that the degree of prevalence (and thus, the degree of accuracy in phase identification) across the different combinations is widely different. We have that the major phases for Ag/Fe, Ag/Cu and Cu/Fe are generally highly prevalent, only decreasing along transition regions. In contrast, the three systems that contain nickel do showcase less defined regions, particularly at alkaline pH. Most of this lack of definition comes from the competition between “free” Ni^2+^, solid NiO (s), the oxo-hydroxide NiO(OH) (aq) and the hydroxides ${\rm{Ni}}{({\rm{OH}})}_{3}^{-}$ and ${\rm{Ni}}{({\rm{OH}})}_{4}^{2-}$, strongly limiting the definition of clear frontiers between these phases. In all cases, the most reductive potentials are completely controlled by solid A(s) + B(s) phases, except for Fe/Ni, where the mixed FeNi(s) is formed instead. In general, the addition of the competition layer to the Pourbaix diagram does immediately reveal relevant chemical processes (e.g., the equilibria established by nickel at neutral and alkaline pH), with minimal overheads in both the calculation of the diagram and its interpretation (calculation time details shown in Fig. S2).

To validate our approach, we compared the predicted phase populations with experimental observations for the Ni − H_2_O system, studied by Huang et al.^23^ through electrochemical impedance spectroscopy (EIS) and Raman spectroscopy. Therein, experimental impedance measurements show maxima precisely at the pH boundary where our Boltzmann populations for the Ni-bearing systems predict low prevalence ( < 70%) and consequent strong phase competition. Notably, Huang et al. also employed Boltzmann-like probability distributions to quantify multi-phase coexistence and to verify the continuous nature of phase boundaries in Pourbaix diagrams, independently validating the statistical framework underlying our approach^23,24^. Thus, the consistency between theoretical predictions and spectroscopic phase identification demonstrates the reliability of our analysis to identify regions where traditional Pourbaix diagrams oversimplify phase behavior.

We can also use a more quantitative analysis to simplify the comparison of multiple Pourbaix spaces, thus defining a consistent approach for the comparative analysis of Pourbaix diagrams. From the same data depicted in Fig. 1, we determined the average and maximum population of each region, its center and its relative area, as mentioned in the “Methods” section. From these results, we discarded the smallest regions (area below 5.0%) for simplicity and brought together the results for the six combinations in the dataset.

**Fig. 2:**
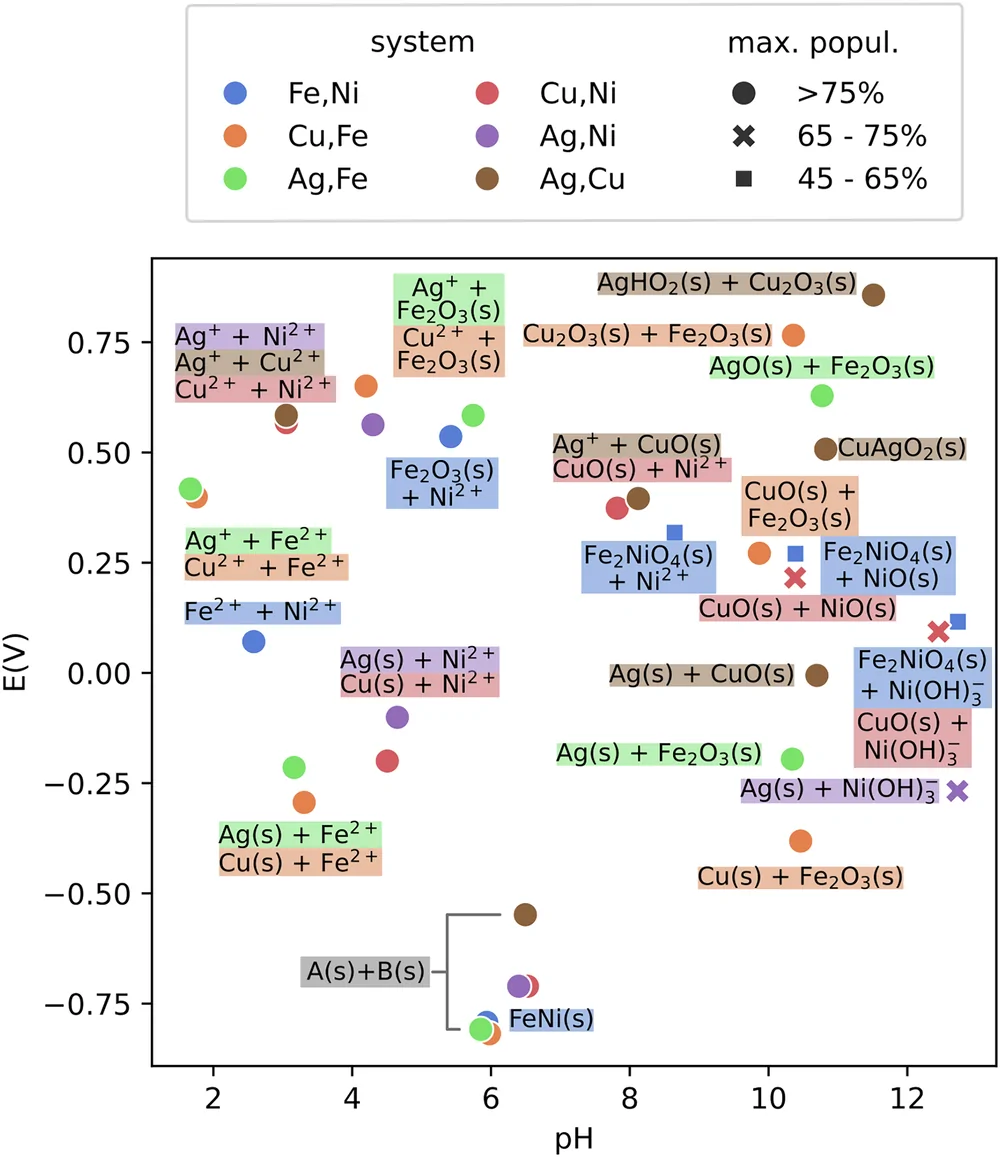
Map of phases occupying more than 5% of the Pourbaix diagram for A/B metal combinations. X (pH) and Y (V) positions correspond to the center of the region. Colors indicate metal combination: Fe/Ni (blue), Cu/Fe (orange), Ag/Fe (green), Cu/Ni (red), Ag/Ni (purple) and Ag/Cu (brown). Qualitative binning of the *maximum* population across the region is depicted with markers: circles for > 75%, crosses for 65–75% and squares for 45–65%.

Figure 2 streamlines the direct comparison between the systems. For instance, the cluster of points at negative potentials highlights the aforementioned prevalence of A(s) + B(s) phases (except for the FeNi(s) alloy) in this region of the diagram. We can also visualize the similarities between Cu/Fe and Ag/Fe, with the corresponding orange (Cu/Fe) and green (Ag/Fe) points across the map appearing in close proximity, regardless of the pH and potential, only differing in the oxidation state of the oxidized metal at large potentials being Ag(I) or Cu(II). At acidic pH, a similar relationship is shown for Cu/Ni (red) and Ag/Ni (purple), although the similarities break down in the alkaline region due to the complex behavior of nickel species at these pH values. While in this particular example we mostly encounter widely known trends (e.g., the analogies between the coinage metals copper and silver, the already observed dominance of solid mixtures and alloys at more reductive potentials), it again aims to demonstrate the versatility of our approach to make the most from the information-rich Pourbaix space that traditionally would only be used to predict individual diagrams. For completeness, speciation diagrams for all six combinations are provided in the Supporting Information (Fig. S3).

### Iron-based alloys

Following the topic of binary metal combinations, we performed a preliminary study on stainless steel alloys to further validate our approach. For simplicity, we considered a simple 80% Fe/20 % Cr austenitic stainless steel model, and explored the corresponding competition-informed Pourbaix diagram (Fig. S7) and speciation diagrams (Fig. S8). In this context, we compare our population analysis with two recent studies on the applicability of Pourbaix diagrams to Fe/Cr alloys^25,26^. The full discussion is available in the Supporting Information (Section S7); here, we just provide a brief summary of the main findings. We should remark that both these studies also consider theoretical Pourbaix diagrams, as no direct experimental validation analogous to that of Huang et al. for Ni^23,24^ was available for Fe/Cr.

First, Muñoz-Portero and co-workers^26^ explore how the phase transition associated with the corrosion of the passivating Cr_2_FeO_4_ layer in a broad potential window (−0.5 to 0.5 V) shows large displacements depending on the ion activity used in the construction of the diagrams. We showcase (Fig. S7) a wide zone with low maximum prevalences, below 50%, where Cr_2_FeO_4_ competes with the ionic species Cr^3+^ and Cr(OH)^2+^—depicting a continuous transition between corrosion and passivation instead of a hard limit. In this context, these extended regions of low CrFe_2_O_4_ prevalence correspond to regions where the passive film is thermodynamically fragile: consequently, to potential windows where localized breakdown (*pitting*) of the oxide layer could take place even if assigned to pure passive phases in the traditional diagram.

Second, Malki et al.^25^ apply systematic free energy perturbations to the information in the Materials Project database to study metastability processes, discussing the extension of the Cr_2_O_3_ domain at alkaline, reductive conditions. Through our approach, we effectively identify relevant competition between Cr_2_O_3_ and the major phase Cr_2_FeO_4_ in this region (Fig. S8), thus confirming the importance of this phase under these conditions.

Consequently, the current preliminary study already demonstrates how the analysis of competition effects can immediately reveal relevant electrochemical trends out of the box for real-world systems, without needing to construct specific analysis pipelines. A future direction we aim to pursue is to expand our study to the more complex ternary Fe/Cr/Ni and quaternary Fe/Cr/Ni/Mo phases, showcasing further applicability of PourbaixUP to systems of industrial relevance.

### Bismuth telluride

Bi_2_Te_3_ is a remarkably important material for thermoelectric applications^3,27,28^, which is often produced by electrodeposition^29,30^ from Bi(III)/Te(IV) solutions. Consequently, understanding the behavior of these mixtures under different electrochemical conditions is essential to guide the development of this kind of materials and devices, as it has been demonstrated^3^ that careful control of electrodeposition conditions (potential and pH) is crucial to achieve the deposition of Bi_2_Te_3_(s), precisely due to the competition of other possible phases. Thus, we chose it as another example to showcase the importance of phase prevalence analysis to enhance the interpretation of Pourbaix diagrams. Following the previously described strategy, we characterized the Pourbaix space of the system and overlaid the prevalence contour plot and the limits of the traditional diagram.

**Fig. 3:**
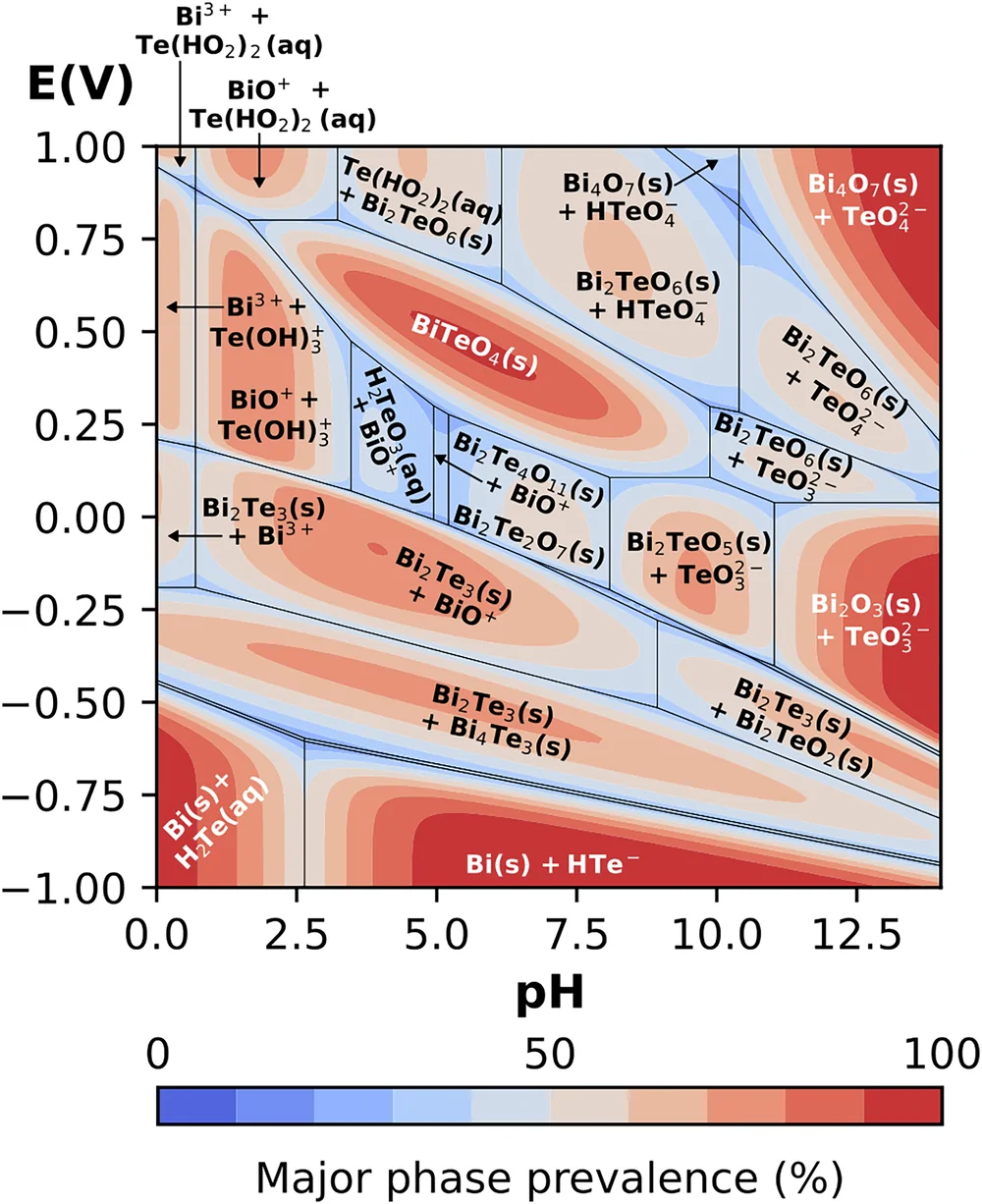
Boltzmann-weighted Pourbaix diagram for the Bi/Te system. 50% Bi, 50% Te, V range (vs. SHE) −1.0 V to 1.0 V, pH range 0 to 14. Color scale corresponds to the population of the major phase, labeled in the graph. Red/warm colors denote single dominant phases ( > 80%), blue/gray colors ( < 70%) denote regions where Boltzmann populations are distributed across multiple phases, indicating strong phase competition due to near-degenerate formation energies.

Figure 3 exemplifies again the appearance of phase transitions as regions of reduced prevalence. Moreover, fundamental differences across different regions in the diagram are also clear. At more negative potentials, we find Bi(s) + TeH_2_(aq) and Bi(s) + TeH^−^, two very prevalent phases that reach almost 100% population and only decay right at the transition. A similar phenomenon occurs to BiTeO_4_(s), in the upper central part of the plot. In contrast, at the upper right part (moderate-high potentials, alkaline pH), Boltzmann populations reveal strong phase competition, as the major phase (${{\rm{Bi}}}_{2}{{\rm{TeO}}}_{6}({\rm{s}})+{{\rm{TeO}}}_{3}^{2-}$ and ${{\rm{Bi}}}_{2}{{\rm{TeO}}}_{6}({\rm{s}})+{{\rm{TeO}}}_{4}^{2-}$) prevalences drop below 70%. Close to the central region, the two-ion phase BiO^+^ + TeH_2_O_3_(aq) has even lower maximum prevalences, highlighting the high phase competitiveness in this part of the pH/V space (neutral pH and small positive voltages).

**Fig. 4:**
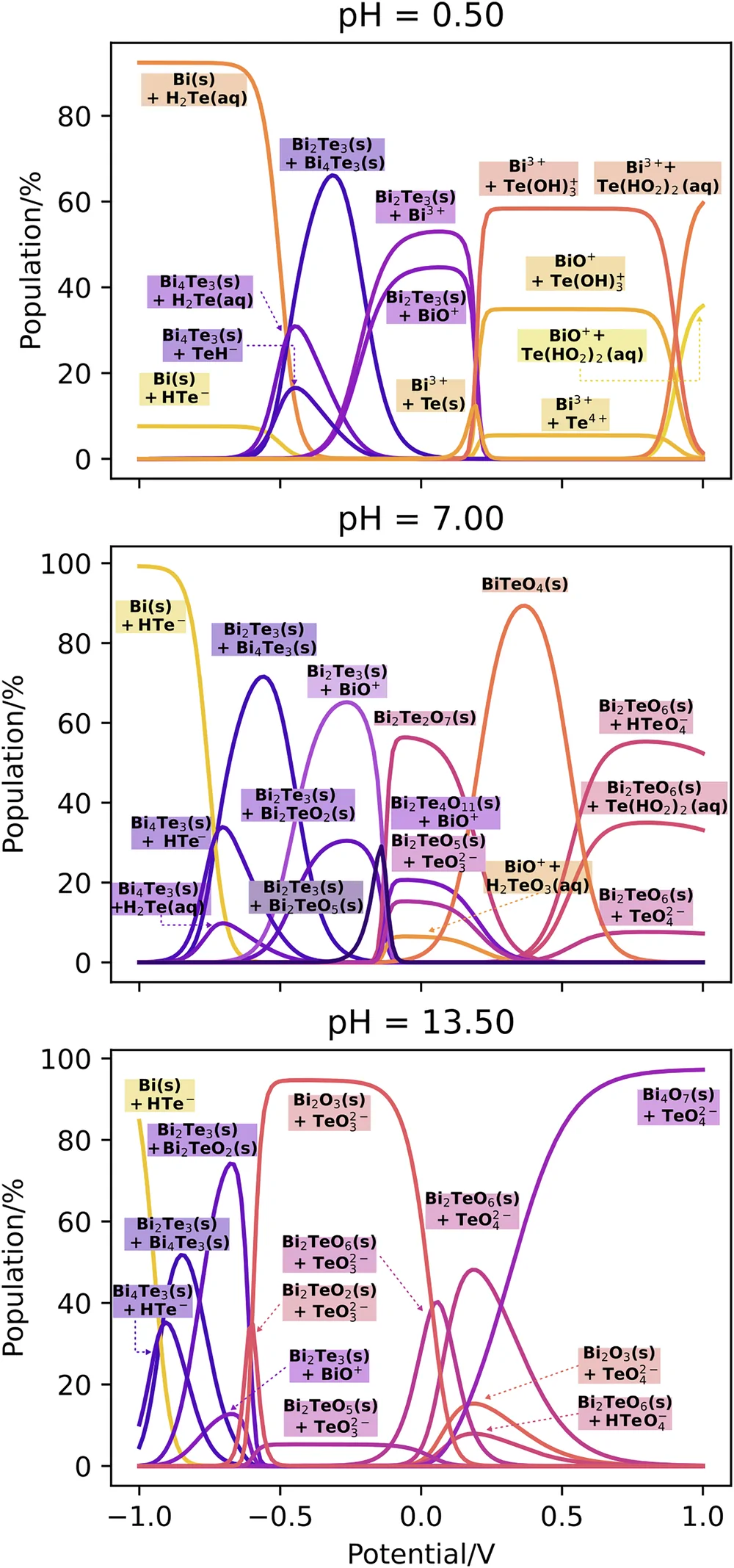
Speciation diagrams for the Bi/Te system. 50% Bi, 50% Te, V range (vs. SHE) −1.0 V to 1.0 V, at pH values of 0.5 (acidic, top), 7.0 (neutral, central) and 13.5 (alkaline, bottom). The color scale is qualitative.

In this way, just like before, our predictions enable us to distinguish phases that would seem equally dominant in a traditional Pourbaix diagram, in spite of clear differences in the likelihood of phase competitiveness. From there, our framework enables us to consider multiple alternative analyses on the obtained data, going *beyond* this more traditional view. For instance, we may consider the *speciation* of the system at fixed pH values, as showcased in Fig. 4.

The analysis of the population vs. potential curves is more convoluted than that of the Pourbaix diagram, but can provide relevant insights for the less defined regions of the pH/potential space, highlighting not only the likelihood of competitive phases arising, but also some clarification on the identity and influence of these competitive phases. Considering the system at acidic pH (top of Fig. 4) at positive potentials, we observe the competition between the Bi^3+^ and BiO^+^ cations in combination with the ${\rm{Te}}{({\rm{OH}})}_{3}^{+}$ ion. This information is not present in the Pourbaix diagram, where we would only assign the ${{\rm{Bi}}}^{3+}+{\rm{Te}}{({\rm{OH}})}_{3}^{+}$ phase. The same Bi^3+^/BiO^+^ interplay happens around *V* = 0, with populations of both phases (coexisting with solid Bi_2_Te_3_(s)) being even closer.

Going to neutral pH, we have a very convoluted situation in the central region (approximately between −0.5 and +0.5 V), with a variety of competing phases that contribute to the overall lack of phase assignment accuracy that was hinted at by the competition-informed Pourbaix diagram (Fig. 3). The speciation diagram shows how this effect comes from the likely coexistence of different solid phases (Bi_2_Te_3_(s), Bi_2_TeO_2_(s), Bi_2_TeO_5_(s), Bi_2_Te_2_O_7_(s), Bi_2_Te_4_O_11_(s), etc), highlighting the sheer complexity of this particular pH and potential window—exactly the kind of case for which a direct assignment of the major phase would encounter limitations.

Important competition between phases is also shown at alkaline pH, coming both from ion (${{\rm{TeO}}}_{3}^{2-}/{{\rm{TeO}}}_{4}^{2-}$ between 0.0 and 0.5 V) and solid interconversions (right above −1.0 V, Bi_2_Te_3_(s), Bi_4_Te_3_(s) and Bi_2_TeO_2_(s) are involved). The overall idea behind these two possible views is for them to be complementary. First, the extended Pourbaix diagram enables the immediate identification of less certain regions. If the conditions associated with these regions are of interest, speciation diagrams at fixed pH (or fixed potential) can be traced to dig deeper into the behavior of the system of choice.

Going a step forward in system complexity, ternary Bi_x_Sb_2−x_Te_3_ combinations have also been widely exploited^31–34^ as thermoelectric materials. Although Pourbaix diagrams can also be easily constructed for this kind of system, the prevalence of the individual major phases reflected in the diagram is indeed remarkably lower than for the non-Sb-doped system.

**Fig. 5:**
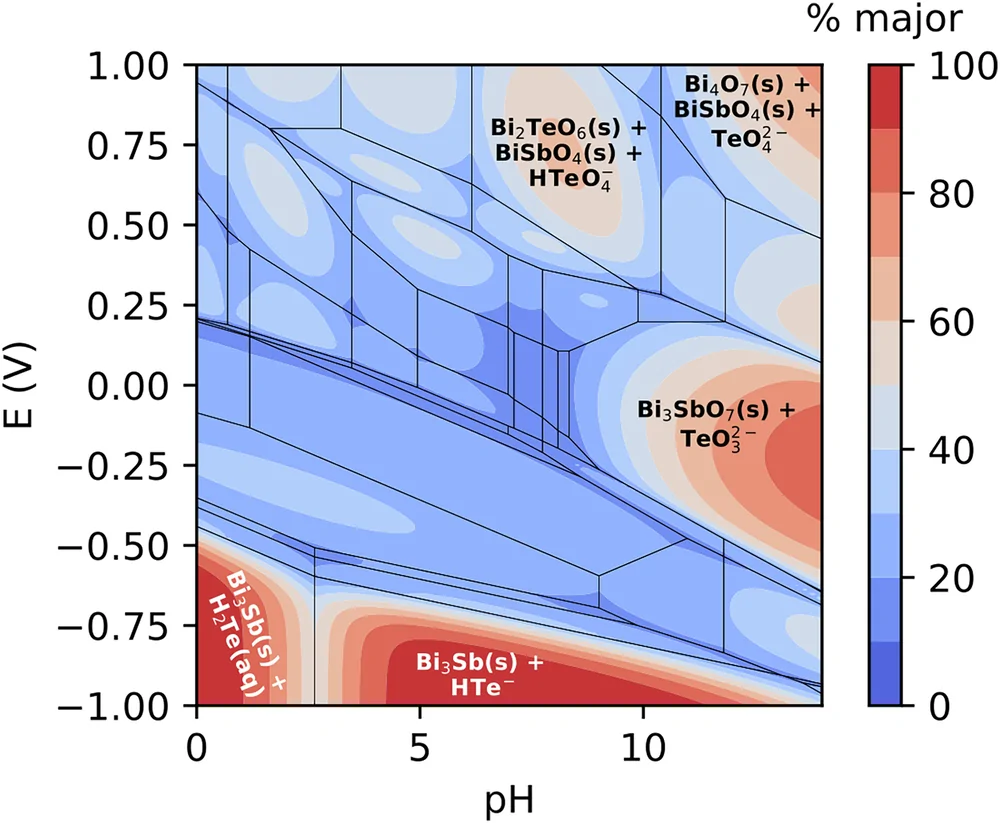
Boltzmann-weighted Pourbaix diagram for the Bi/Sb/Te system. 30% Bi, 10% Sb, 60% Te, V range (vs. SHE) −1.0 V to 1.0 V, pH range 0 to 14. The color scale corresponds to the population of the major phase. Red/warm colors denote single dominant phases (>80%), blue/gray colors ( < 70%) denote regions where Boltzmann populations are distributed across multiple phases, indicating strong phase competition due to near-degenerate formation energies. Labels only included for phases with maximum prevalence above 60%.

We considered **x** values between 0.0 and 2.0, and computed the populations with the same (pH, V) grid as in the previous examples. We showcase an example of the resulting expanded Pourbaix diagram for a mixed-phase Bi_1.5_Sb_0.5_Te_3_ in Fig. 5.

The diagram in Fig. 5 shows a large degree of phase competition, except for the specific regions between −0.5 and −1.0 V (Bi_3_Sb(s) + tellurium hydrides) and the patch corresponding to Bi_3_SbO_7_(s) at highly alkaline pH. This is a natural consequence of the increase in the complexity of the system, as more species enter the competence due to the interplay between bismuth, antimony and tellurium. It also highlights the limitations that traditional Pourbaix diagrams may face when dealing with complex mixtures, as the assignment of the dominant phase is likely to become less obvious.

From Fig. 5, we can easily identify the pH/potential windows where the major phase assignment is more diffuse and explore them in more detail. Following our previous strategy, we might consider speciation diagrams at fixed potential or pH—however, they will also be even more complex than those in Fig. 4, complicating their representation and interpretation. Moreover, as discussed in the “Methods” section, modifying the composition in our Materials Project-based calculation framework not only alters the phase populations but also the *selection* of the involved phases. Therefore, the comparison of speciation or Pourbaix diagrams with varying initial proportions of Bi and Sb would be hindered by the participation of widely different phases in each Pourbaix space. To tackle these issues, we consider an alternative dimensionality reduction focusing on *composition* and *population*, fixing the potential and pH values—which would be equivalent to analyzing a fixed (x,y) point in an ensemble of 3D Pourbaix spaces at different compositions. We represent this analysis with a bar plot with the populations of the main species at *V* = 0.0 V and pH = 7.0 (where Fig. 5 is less defined), showing the evolution of these major phases with the amount of bismuth.

**Fig. 6:**
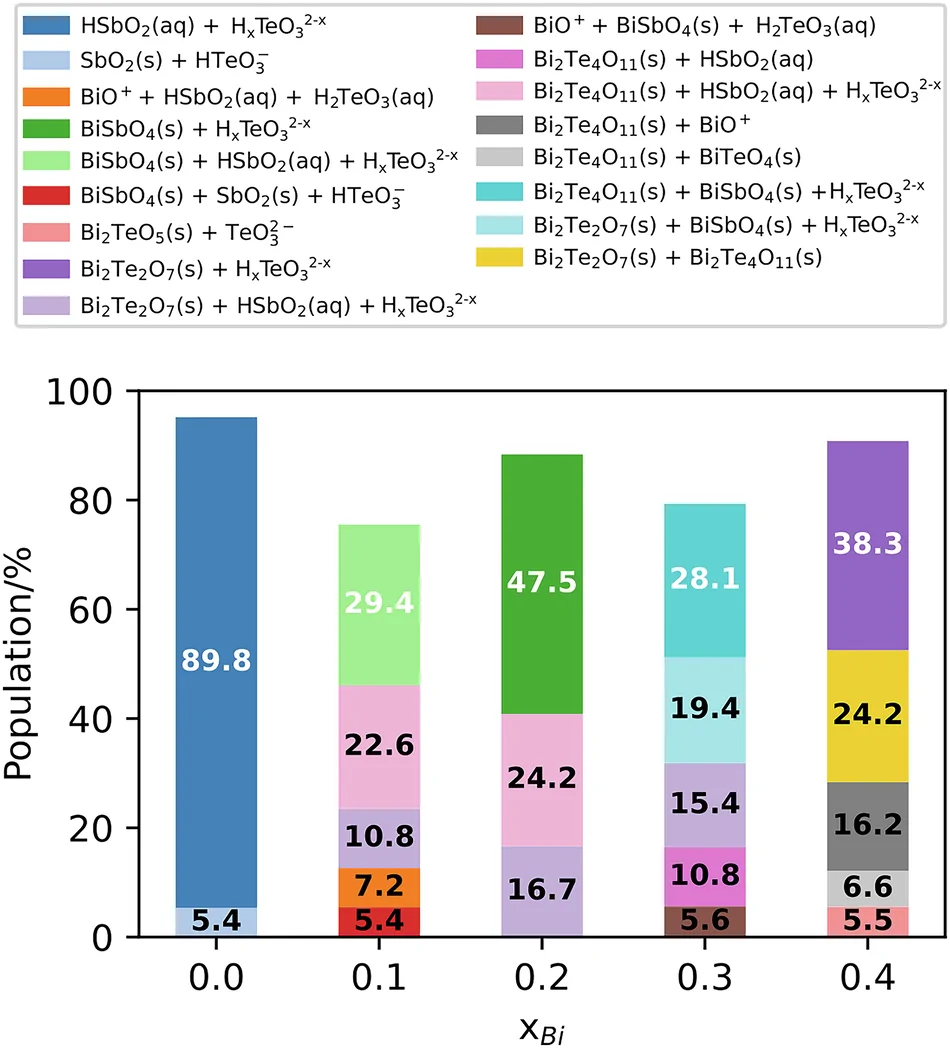
Main speciation of Bi/Sb/Te system at pH = 7.0 and *V* = 0.0 V (vs. SHE), with varying amounts of Bi (0.0 to 0.4). Only the 15 major phases and those with populations above 5.0 % are included, leading to the total population being lower than 100%. Phases that only differ in the protonation state of tellurite (${{\rm{H}}}_{{\rm{x}}}{{\rm{TeO}}}_{3}^{2-{\rm{x}}}$) are collated together. The color scale is qualitative.

Figure 6 showcases a direct comparison between systems with varying Bi/Sb amounts, which will not be possible at all with traditional Pourbaix diagrams—demonstrating, again, the relevance of our methodology. We see how for Sb_2_Te_3_ (*x*_*Bi*_ = 0.0) the ionic phase containing tellurite and HSbO_2_ is clearly dominant under these conditions; nonetheless, when Bi is present, solid phases take the lead. The mixed oxide BiSbO_4_(s) is somehow dominant in the mixtures, in coexistence with phases where excess Bi forms oxo-tellurides and Sb remains as HSbO_2_(aq). For *x* = 0.4, Bi_2_Te_2_O_7_(s) is the most prevalent phase, in alignment with Pourbaix and speciation diagrams (in spite of the compositional change between previous examples, with Bi:Te proportions being 0.5:0.5 instead of 0.4:0.6 like here). Apart from the *x* = 0.0 case, none of the phases reaches a 50% prevalence, remarking the high degree of competition taking place for the ternary system under these conditions.

Analogous plots for acidic (pH = 0.5) and alkaline (pH = 13.5) media, still at *V* = 0.0 V, are shown in the Supporting Information (Figs. S4 and S5). For acidic media, the overall competition is mostly due to the ionic equilibrium HSbO_2_ *⇌* SbO^+^ + OH^−^, as solid phases are dominated by Sb_2_Te_3_(s), Bi_2_Te_3_(s) and their mixtures. For alkaline media, the stoichiometric composition *x*_*Bi*_ = *x*_*Sb*_ = 0.2 shows almost total prevalence for ${{\rm{BiSbO}}}_{4}({\rm{s}})+{{\rm{TeO}}}_{3}^{2-}$, while the other compositions have several oxides and mixed Bi/Sb oxides in competition.

Yet again, it is important to emphasize that these population percentages should not be interpreted as physical concentrations (amounts) of the involved phases—that is, the values in Fig. 6 do not mean that, for instance, exactly a 47.5% of ${{\rm{BiSbO}}}_{4}({\rm{s}})+{{\rm{H}}}_{{\rm{x}}}{{\rm{TeO}}}_{3}^{2-{\rm{x}}}$ should be measurable at *V* = 0.0 V and pH = 7.0 for a Bi_0.2_Sb_0.2_Te_0.6_ mixture. Instead, this value quantifies a moderate-to-low confidence in assigning this phase to this region of the pH/potential space, showcasing the importance of taking into account the competing phases (involving the solids Bi_2_Te_4_O_11_(s) and Bi_2_Te_2_O_7_(s)) to understand the system—meaning that the predictions of the method should only be taken as *semiquantitative* results.

With this example, we have returned to the key question of reducing the *dimensionality*—incorporating the composition transforms the already defined 3D Pourbaix space into a 4D space (pH/potential/population/composition) that necessarily requires fixing some of the variables to be represented. In this case, we fix pH and potential, losing the “bigger picture” painted by the individual Pourbaix diagrams, to focus on the intimate behavior happening at specific conditions. This circles back to the fundamental idea behind our whole approach, which was the intrinsic dimensionality reduction associated with the selection of the lowest-energy phase for the construction of Pourbaix diagrams. All in all, our proposal aims to provide the conceptual framework and tools to deal with this dimensionality in a more thorough manner, effectively enhancing the applicability of computationally-derived phase diagrams.

From there, a future direction that we aim to pursue is directly coupling competition-informed Pourbaix diagrams and speciation diagrams with experimental setups to follow electrochemical reactivity *in operando*, obtaining spectroscopic fingerprints of the species that are produced at varying pH/potential conditions. Apart from a further direct confirmation of the relevance of phase competitiveness, such a combined approach directly links with the core notion of *rational material design*, using Pourbaix spaces as a versatile tool to interpret and predict complex phenomena.

### Overview

We present a framework based on the calculation of Boltzmann-like populations to enrich the computational prediction of Pourbaix diagrams with competition information, defining *Pourbaix spaces* that characterize phase competitiveness semiquantitatively across the pH and potential ranges used for diagram generation. In this context, we determine the percentual population of the major phase at every (pH,V) point across the diagram as the *prevalence* of that phase and therefore as the *accuracy* of the traditional assignment. This enables the direct characterization of the regions of the phase diagram where the competition between phases might alter the prediction of the dominating one, and thus the actual electrochemical processes happening in the system. Moreover, this representation also naturally depicts the phase transition regions, where the major phase switches.

Going beyond the direct inspection of these major phase prevalences (competition-informed Pourbaix diagram), we also propose the use of speciation diagrams, fixing either the pH or the potential, to reduce the dimensionality of the Pourbaix space and enhance the understanding of the phase competition at conditions of interest.

Additionally, we also introduce a quantification of the geometric regions of the phase diagram, determining their areas and average/maximum population values, to simplify the complex inter-system comparison of multiple Pourbaix diagrams.

The present analysis focuses on metal/oxygen/hydrogen equilibria in pure aqueous solutions at ambient temperature, without considering either the effect of elevated temperatures or the contribution of anions like sulfate or chloride. While these factors can be important in processes such as corrosion, the current Materials Project-based framework cannot take their effect into account explicitly yet.

To demonstrate the versatility of these advancements, we explore three relevant use cases, such as transition metal binary combinations of Ag, Cu, Fe and Ni, Fe/Cr alloys in the context of stainless steel corrosion and chalcogenide materials like bismuth and bismuth-antimony tellurides, of relevance for thermoelectric applications. For the metal combinations, our approach directly reveals the influence of the equilibria between nickel (II) oxide and its hydroxides in alkaline media, inducing competitiveness in the corresponding regions of the diagram. Moreover, we also highlight the strong similarities between different metal pairs, such as Ag/Fe and Cu/Fe, across changes in pH and potential.

For the corrosion studies, we contextualize trends reported in the literature, such as the displacement of FeCr_2_O_4_ dissolution lines and the influence of Cr_2_O_3_ at alkaline pH and highly reductive potentials in terms of interphase competition, avoiding the need to generate and compare multiple Pourbaix diagrams under varying conditions.

For the chalcogenides, we showcase the complexity of the Bi/Te system and the existence of phases of clearly distinct prevalences, particularly at regions where the pH is close to neutrality and the potential is close to zero. Speciation diagrams shed light on these more complex regions, exploring the evolution of the involved phases with the potential at fixed values of pH (acidic, neutral and alkaline). Finally, we apply the same approach to a ternary combination, Bi/Sb/Te, testing different initial compositions of bismuth and antimony. This illustrates the importance of competitiveness characterization for systems of increasing complexity, with much less defined phases through most of the pH/V space.

Finally, we also distribute an open-source software package, PourbaixUP, containing all the developed procedures for the calculation of populations from the data available in the Materials Project (Pourbaix space generation) as well as for their further analysis. Moreover, we also provide an interactive interface to simplify the generation of extended Pourbaix diagrams and speciation diagrams for less-experienced users.

## Methods

### Background

To compute the free energy of each phase in the Pourbaix diagram, we follow the methodology employed by the *pymatgen* package from the Materials Project^10,13^, defining a grand thermodynamic potential^12,35^, introducing the voltage (V) and the chemical potential of the proton (*μ*_*H*_) that can then be transformed to a pH-dependent term. Then, we obtain an expression (Equation (1)) involving the free energy of the phase (*G*_*phase*_) at *V* = 0 and pH = 0, the potential and the pH, and the numbers of acidic protons *N*_*pH*_ and electrons *N*_*ϕ*_. A prefactor *f* = (ln10)*k*_*B*_*T*, is also introduced, taking the value *f* = 0.0591 eV at ambient temperature. The energy is finally normalized by the number of non-hydrogen and non-oxygen atoms *N*_*non*−*HO*_, as the Materials Project electrochemical database regards aqueous environments and is therefore dominated by oxides and protonated oxides, with all other atoms being conserved upon transformations^12^. All potentials are referenced to the Standard Hydrogen Electrode (SHE).

Additional details on the derivation of Equation (1) (from refs. ^12,35^) are reported on the Supporting Information.

1 $$\bar{\Psi }({\rm{pH}},{\rm{V}})=\frac{{G}_{phase}+f\cdot {N}_{pH}\cdot {\bf{pH}}+{N}_{\phi }\cdot {\bf{V}}}{{N}_{non-HO}}$$

Considering the exponential dependence between concentrations or activities (in general, *populations*) and energies, employing energy values directly to evaluate the prevalence of the phases would be problematic. We consequently considered Boltzmann-like distributions, following usual procedures for the treatment of thermodynamic equilibria, to define the population of each phase, computed as percentages (Equation (2)):

2 $${P}_{i}=\left(\frac{{e}^{-{\bar{\Psi }}_{i}({\rm{pH}},{\rm{V}})/{k}_{B}T}}{{\sum }_{j}{e}^{-{\bar{\Psi }}_{j}({\rm{pH}},{\rm{V}})/{k}_{B}T}}\right)\cdot 100$$

To illustrate the scope of this work, from Equation (2), we can consider a situation with two phases whose energy difference is comparable to the thermal energy *k*_*B*_*T*: ${\bar{\Psi }}_{0}$ and ${\bar{\Psi }}_{0}+{k}_{B}T$. At 298.15 K, this will correspond to an energy difference of around 25.7 meV (well within the DFT error range 10–100 meV/atom) and to a population split 74:26—which a traditional analysis will neglect, assuming a 100:0 split instead. Consequently, we can qualitatively link the magnitude of DFT errors with thermal energy differences, showcasing how our scheme indicates thermodynamic near-degeneracy. A more detailed example is provided in Section 2 of the Supporting Information (Table S1 and Fig. S1).

Although the *pymatgen* module computes the limits of the Pourbaix diagram by intersecting the corresponding hyperplanes, population calculations must be done in a *grid* of potential and pH values. Nonetheless, the simplicity of Eqs. (1) and (2) enable a very fast calculation for relatively large grids (150 × 150) that already provide a good degree of detail (see Supporting Information, Fig. S2 for timing discussion and Fig. S6 for grid size exploration). These populations should not be regarded as an explicit quantification of the amount of each phase that would be found in solution, but as a *semiquantitative* diagnostic metric to determine the degree of competitiveness and the accuracy of the traditional Pourbaix diagram assignment in that region. That is, regions where Boltzmann populations are distributed across multiple phases, with formation free energy differences from the lowest-energy phase being around *k*_*B*_*T* (i.e., also within the DFT uncertainty window), are precisely those where the assignment of a single dominant phase becomes unreliable.

Evaluating Equations (1) and (2) provides the populations of each of the phases in the system at every point in the grid—thus, at every (pH,V) combination across the diagram. From now on, and for simplicity, we will label this 3D space (population/pH/potential) as a *Pourbaix space*. We can then easily determine the *major* phase at every point, which is the one represented in the Pourbaix diagram. From the *actual* population of the major phase, we can showcase how prevalent these different phases are across the diagram, effectively building a competition-informed Pourbaix diagram. Due to the generality of this strategy, future extensions might target the integration of energy correction schemes like the ones stated in the Introduction^15,20^ or even the application of the same procedure to other kinds of phase diagrams beyond the Pourbaix. In this line, it is also important to remark that, of course, the reliability of any Pourbaix-space-based analysis is dependent on the reliability of the underlying data in the Materials Project database. Thus, if the energies or phases reported for a given system are inadequate, neither the traditional Pourbaix diagram nor the present population-based analysis will provide realistic predictions—requiring the direct curation of the dataset.

Furthermore, all Pourbaix spaces explored in this work consider a temperature of 298.15 K, introduced in the prefactor *f* present in Equation (1) as predefined in *pymatgen*. Extension to higher temperatures, of relevance in e.g., corrosion processes, will be integrated into PourbaixUP in the future.

### Data representation

As the core of this work is facing the dimensionality reduction of the tridimensional Pourbaix space, we can also explore the phase distribution in different ways by choosing alternative ways to tackle this reduction. Instead of visualizing simultaneous changes in pH and potential, as in the Pourbaix diagram, we may fix one of the variables, considering a buffered solution (fixed pH) or a constant-potential electrochemical setup. Then, we can visualize the changes in the population of all available phases against the non-fixed variable (V or pH)—constructing speciation diagrams. This approach is in line with previous work on the prediction of the aqueous self-assembly of metal-oxo clusters^36–39^. These studies also considered a 3D space, but with different variables, having the total concentration of the initial metal cluster instead of the electric potential. From there, both composition vs. pH (speciation diagram, at a fixed metal concentration) and maximum composition vs. metal concentration and pH (phase diagram, analogous to the Pourbaix) were represented. It is worth noting that Pourbaix diagrams for similar metal-oxo clusters (Al, Ga) have also been computationally explored from data in the Materials Project^40^. Again, the populations in the current framework should not be aligned to actual phase concentrations, as the reliable characterization of these would require a profound reinterpretation of results, considering the *statistical weights* of each combined phase. In the context of Pourbaix spaces from the Materials Project, a mixed A + B + C phase does *not* imply the contribution of each component to be equivalent: thus, the mixed phase is not 1/3 A + 1/3 B + 1/3 C, but more generally aA + bB + cC, with (a,b,c) being the aforementioned weights. This additional complexity would hinder the main advantage of our current approach, both in terms of competition-informed diagrams and speciation diagrams, which is the immediate enrichment of traditional Pourbaix analysis with phase competitiveness information. This is a factor that is completely neglected in traditional analyses, and thus its semiquantitative characterization is already a valuable asset.

**Fig. 7:**
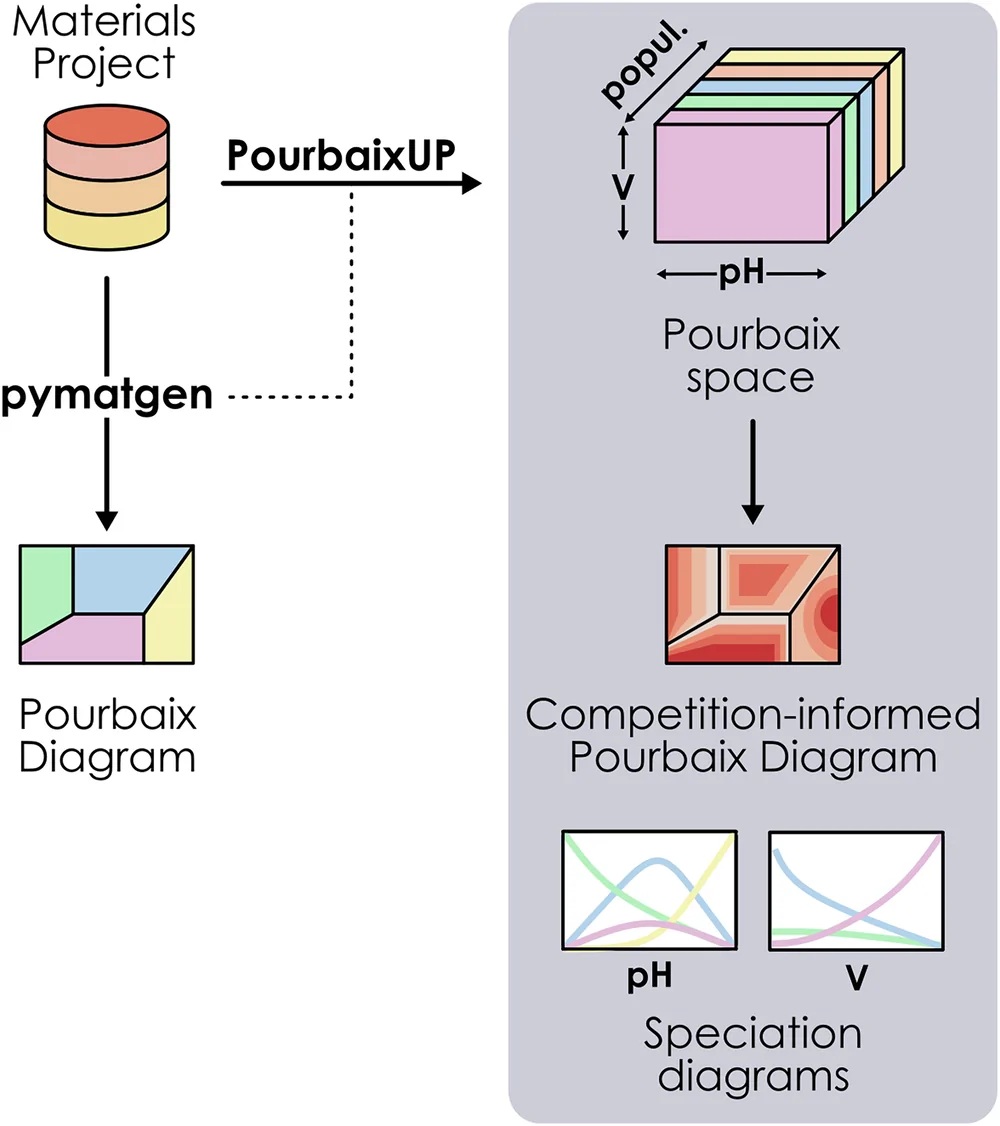
Schematic depiction of the data flow and main kinds of plots generated by the proposed strategy (PourbaixUP).

The general data management procedure, leading to either competition-informed Pourbaix diagrams or to speciation (population vs. pH or population vs. potential) diagrams, is illustrated in Fig. 7.

Previous efforts over Materials Project-based Pourbaix diagrams have already considered a somewhat similar concept, exploring the decomposition energy of individual phases exploring possible *metastability* regions over the diagram^12^. While the relationship is clear, there is a fundamental difference with the current approach, as the metastability characterization focuses on one phase at a time, while ours provides a general, “big picture” overview of the system, highly relevant for exploratory analyses on systems containing many phases. On the other hand, a similar proposal was introduced by Huang and co-workers^23,24^, introducing *population profiles* based on Boltzmann probabilities, analogous to our speciation diagrams, to improve the analysis of transition regions. Nonetheless, this approach was not directly connected with the Materials Project database nor directly explored the consideration of phase prevalences over the Pourbaix diagram.

Expanding the previous concepts, we can also manipulate the Pourbaix space (population/pH/potential data) to obtain numeric descriptors for the different regions. These involve magnitudes such as the *extension* of the region (number of points occupied by a phase divided by the total number of points in the grid), its average or maximum population, the standard deviation, etc. In this way, we can build a quantitative framework to easily compare a set of Pourbaix diagrams for different systems.

For multi-element mixtures, we can also define the initial *composition* of the mixture, which controls which mixed phases can actually appear. As discussed by Patel et al.^12^, the current implementation of multi-phase definition in *pymatgen* considers a convex-hull-based approach to accelerate the construction of many-element mixtures. Thus, only the entries falling inside the convex hull for a given composition are considered in the Pourbaix diagram calculation; these are the phases for which we build the Pourbaix space and compute populations. For instance, we can consider an M_2_X_3_ metal/chalcogenide system (e.g., Bi_2_Te_3_). At the exact stoichiometric ratio M/X 0.4/0.6, a “pure” M_2_X_3_ phase will be stable and appear in the diagram. At any other ratio, the feasible phases must accommodate the excess of metal or chalcogen (e.g., M_2_X_3_ + M^3+^)—thus, not only the differences in the populations, but also the change in the actually accessible multi-species phases must be taken into account. With this in mind, we may also tackle the evolution of the major phase prevalences with varying compositions, effectively adding a fourth variable (composition) to the predictable phase space.

All of these features have been included in a Python package, PourbaixUP, available on the following GitLab repository^41^. PourbaixUP interfaces *pymatgen* to construct the Pourbaix diagrams and extract the corresponding free energies, and then performs the population calculations and analysis. The package also provides an interactive dashboard interface to simplify the adoption of the tool by less experienced users, requiring minimal coding.

## Supplementary information

Supplementary Information

## Data Availability

The data used in the manuscript (binary metal phases, Bi/Te and Bi/Te/Sb systems) was directly extracted from the Materials Project database^13^ using its Python API. An example of the scripts used for data extraction is also available in the GitLab repository.

## Code availability

All the code employed to compute phase populations, competition-informed Pourbaix diagrams and speciation diagrams has been packaged as a Python tool, PourbaixUP, available at https://gitlab.com/dgarayr/pourbaixup. An interactive Streamlit-based web application (Pourbaix Explorer) is also available in the repository to simplify the initial interaction with the program.
