# Supplementary material for: Enriching Pourbaix diagrams for improved understanding of competitive electrochemical phases
Source: NPJ Comput Mater. 2026 Jul 22;12(1):386. doi: 10.1038/s41524-026-02232-5 (PMC13754564; doi:10.1038/s41524-026-02232-5)
Supplement: Supplementary file 1 [file 41524_2026_2232_MOESM1_ESM.pdf]

Supporting Information for: Enriching Pourbaix Diagrams for Improved Understanding of Competitive Electrochemical Phases

Diego Garay-Ruiz[a,*] and Marisol Martín-González[a,*]

[a]Instituto de Micro y Nanotecnología, IMN-CNM, CSIC (CEI UNAM+CSIC), Isaac Newton, 8, 28760, Tres Cantos, Spain

Contents

1 Details on the Pourbaix potential **2**

2 Worked example of phase prevalences **3**

3 Computational overhead **4**

4 Speciation of metal alloys **5**

5 Speciation of Bi/Sb/Te mixtures **5**

6 Grid sensitivity **6**

7 Application to stainless steel alloys **8**

1 Details on the Pourbaix potential

Following the work by Sun et al.[1] as implemented in the *pymatgen* library, the free energy used to build the Pourbaix diagrams, defined as Ψ, is constructed by a Legendre transformation over the Gibbs free energy, considering the chemical potentials (μ_i) and number of atoms (N_i) for O and H, as well as the electric potential (V).

The following relationships are considered: $(\partial U/\partial Q) = V$, $(\partial U/\partial N_O) = \mu_O$ and $(\partial U/\partial N_H) = \mu_{H^+} - V$ (considering μ_{H+} at 0 V potential vs. the SHE).

$$\Psi = G - \left(\frac{\partial U}{\partial N_H}\right) \cdot N_H - \left(\frac{\partial U}{\partial N_O}\right) \cdot N_O - \left(\frac{\partial U}{\partial Q}\right) Q = \\ G - (\mu_{H^+} - V) \cdot N_H - \mu_O \cdot N_O - V \cdot Q \tag{S1}$$

Considering the water - oxygen equilibrium in solution $H_2O \leftrightarrow 1/2O_2 + 2H^+ + 2e^-$, we can substitute the chemical potential of oxygen for the one for water $\mu_{H_2O} = \mu_O + 2\mu_{H^+} - 2V$:

$$\Psi = [(G - N_O \cdot \mu_{H_2O}) + (2N_O - N_H)\mu_{H^+} - (2N_O - N_H + Q) \cdot V] \tag{S2}$$

From Supplementary Equation S2, we should expand two terms: the Gibbs free energy of the phase (G) and the chemical potential of protons μ_{H^+}. For the first, we consider the activity a_i of the phase (unity for solids, concentration-dependent for ions): $G_i = \Delta G^0_{f,i} + RT\ln[a_i]$, where $\Delta G^0_{f_i}$ is the formation energy of the phase, as available on the Materials Project database (formation being considered from oxygen and hydrogen gas).

For the second, we relate with the pH by means of $[H^+] = \exp(\mu_{H^+}/RT)$, obtaining $\mu_{H^+} = -RT(\ln 10) \cdot pH$. For consistency, we substitute the natural logarithm in the previous expression for G_i by the decimal $G_i = \Delta G^0_{f,i} + RT(\ln 10)\log[a_i]$. From there, we arrive to:

$$\Psi = (\Delta G^0_f + RT(\ln 10)\log[a_i] - N_O \cdot \mu_{H_2O}) \\ + (2N_O - N_H) \cdot (-RT(\ln 10)\mathrm{pH}) - (2N_O - N_H + Q) \cdot V \tag{S3}$$

From there, in *pymatgen*, the $RT(\ln 10)$ term is computed as a prefactor at 298.15 K. The ideal gas constant is considered in eV/K ($R = k_B \cdot N_A/q_e$) leading to $f = k_B/q_e \cdot T(\ln 10) = 0.0591$ eV. The first term, including formation energy, activity and the chemical potential of water, is computed as the *energy* of the phase. For the other terms, knowing the composition of each phase, the values of $N_H - 2N_O$ (number of acidic protons in the reaction, N_{pH}) and $N_H - 2N_O - Q$ (number of electrons, N_ϕ) are also computed.

Finally, the reported energies are normalized by the number of atoms distinct from oxygen and hydrogen ($N_{non-HO} = N_{atoms} - N_H - N_O$), providing the final expression:

$$\bar{\Psi} = \frac{G_{phase} + f \cdot N_{pH} \cdot \mathbf{pH} + N_\phi \cdot \mathbf{V}}{N_{non-HO}} \tag{S4}$$

2 Worked example of phase prevalences

Following the example in the Methods section, we provide a worked out example of the extent of how energy differences in the range of the thermal energy k_BT are reflected in the calculation of Boltzmann populations.

Let us consider a four-phase system, with the following free energies $\bar{\Psi}_i$ at a given (pH,V) point in the Pourbaix space:

A:	$\bar{\Psi}_0$
B:	$\bar{\Psi}_0 + k_BT$
C:	$\bar{\Psi}_0 + 2 \cdot k_BT$
D:	$\bar{\Psi}_0 + 10 \cdot k_BT$

Table S1: Energies of the toy system containing A, B, C and D phases, expressed in terms of the lowest energy plus an additional term related to the thermal energy.

A traditional Pourbaix diagram here will only select **A** as the major phase, without considering any contribution of B, C or D. If we expand the normalization term in the denominator of Equation 2 from the main text, we obtain:

$$X_{norm} = \sum_{i=0}^{N} e^{-\bar{\Psi}_i/k_BT} = \left(e^{-\bar{\Psi}_0/k_BT} + e^{-(\bar{\Psi}_0+k_BT)/k_BT} + e^{-(\bar{\Psi}_0+3k_BT)/k_BT} + e^{-(\bar{\Psi}_0+10k_BT)/k_BT}\right) = \\ = e^{-\bar{\Psi}_0/k_BT} \cdot \left(1 + e^{-1} + e^{-3} + e^{-10}\right) \quad \text{(S5)}$$

From there, we can explicitly compute the populations of each phase through Equation 2. Considering that all energies are of the form $\bar{\Psi}_i = \bar{\Psi}_0 + z \cdot k_BT$:

$$P_i = 100 \cdot \frac{e^{-(\bar{\Psi}_0+z\cdot k_BT)/k_BT}}{X_{norm}} = 100 \cdot \frac{e^{-\bar{\Psi}_0/k_BT \cdot e^{-z}}}{e^{-\bar{\Psi}_0/k_BT}(1 + e^{-1} + e^{-3} + e^{-10})} = \frac{100 \cdot e^{-z}}{(1 + e^{-1} + e^{-3} + e^{-10})} \quad \text{(S6)}$$

And therefore we obtain population percentages: A, 70.5 %, B, 25.9 %, C, 3.5 %, D, 0.0 %. As discussed in the main text, assuming a complete dominance of phase A (that is, doing a traditional Pourbaix assignment) is an oversimplification of the system, as the contribution of B+C is non-negligible. Energy differences in the range of the thermal energy do substantially affect the population distribution, which gets far from the 100:0:0:0 split assumed in Pourbaix assignment. Simultaneously, we also observe that when the energy differences become larger, the influence of the less stable phases on the distribution disappears: the influence of D (at 10 times the thermal energy) is negligible, while C (at $+3k_BT$) does only account for a 3.5%. This confirms, as expected, that traditional Pourbaix assignments are accurate when there are no near-degenerate phases.

In this line, and coming back to the simpler two-phase example from the main text, we can explore the explicit effect of the phase energy difference. Regarding $\bar{\Psi}_A = \bar{\Psi}_0$ and $\bar{\Psi}_B = \bar{\Psi}_0 + z \cdot k_BT$, we can directly plot the populations of both phases depending on the energy difference $z = \Delta\bar{\Psi}/k_BT$.

Figure S1 illustrates again that even in a very simple two-phase system, the naive consideration of a minimum-energy phase as the only dominant one can easily break down if the relative differences in the formation free energy are not considered: the minor phase is above 20% prevalence up until $\Delta\bar{\Psi}/k_BT = 1.4$, and above 10% until $\Delta\bar{\Psi}/k_BT = 2.2$. As DFT errors are on the order of magnitude of this thermal energy (at 298.15 K, $k_BT = 25.7$ meV, with a base range being between 10 - 100 meV/atom), these Boltzmann populations can be a reasonable indicator of the points of the pH/potential space in which these DFT errors might affect the prediction — and, consequently, of the regions where the assignment is accurate.

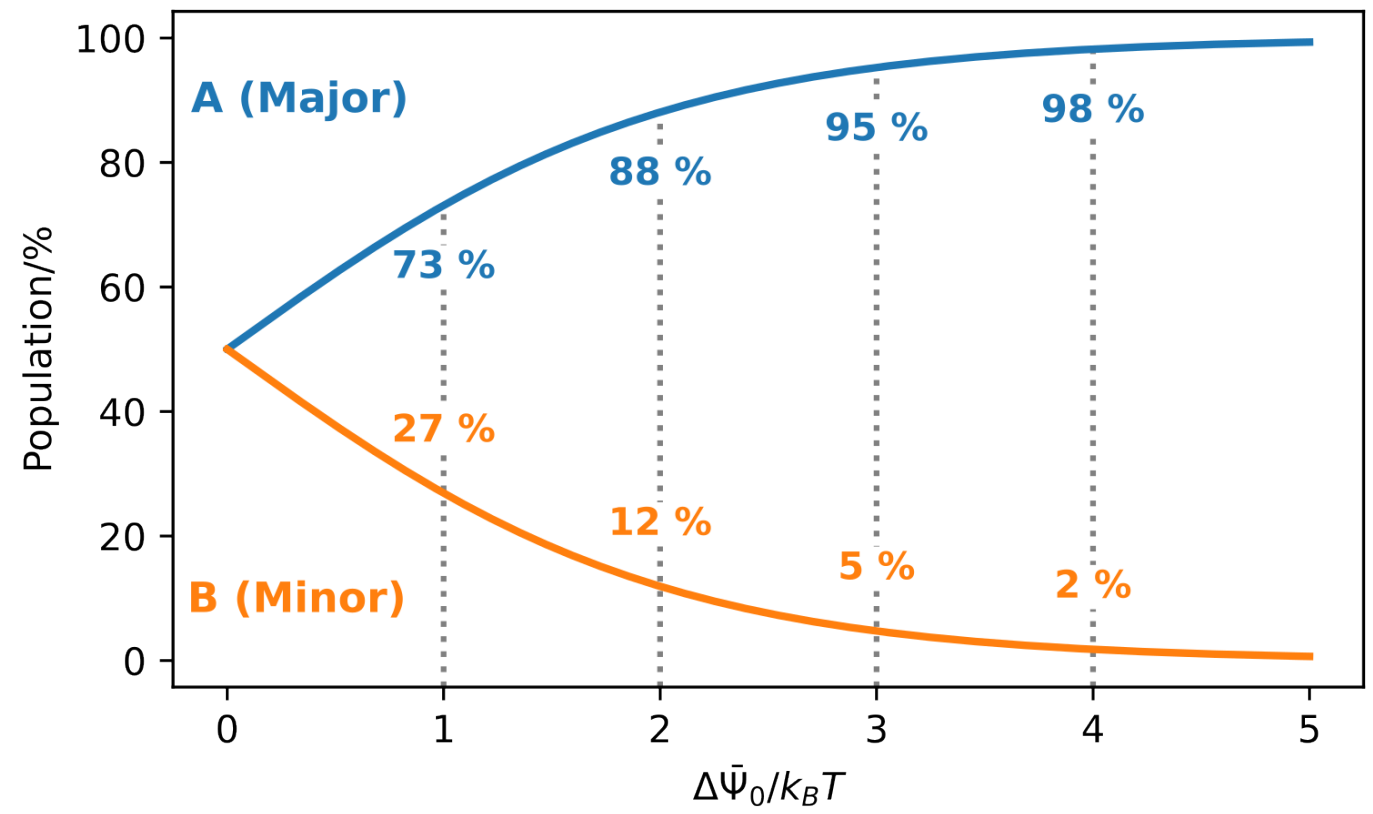

Figure S1: Hypothetical phase populations for A and B with varying energy differences in terms of k_BT. Integer amounts are marked with dotted lines, showing the percentage value of each phase.

3 Computational overhead

To easily characterize the overhead associated to our methodology, we timed the generation of Pourbaix diagrams via pymatgen as well as our population calculation procedure, for a total of 39 phases involving combinations of k transition metals (Ag, Cu, Ni and Fe, as discussed in the main text, k = 1 - 4) as well as a set of binary combinations of possible interest as thermoelectric materials (selenides, tellurides and germanides of Bi, Ag, Cu, Sn, In, Co, Pb and Si).

It must be noted that our strategy *includes* the pymatgen-based calculation of Pourbaix diagrams, in order to leverage the compositional filters available in pymatgen. Thus, the actual calculation time for an extended Pourbaix diagram would be the sum of the time required for Pourbaix diagram processing and the time spent in the grid-based population calculation.

To ensure consistency in the benchmarking, we carried out 10 replicas, for a total of 390 phase population + Pourbaix diagram calculations.

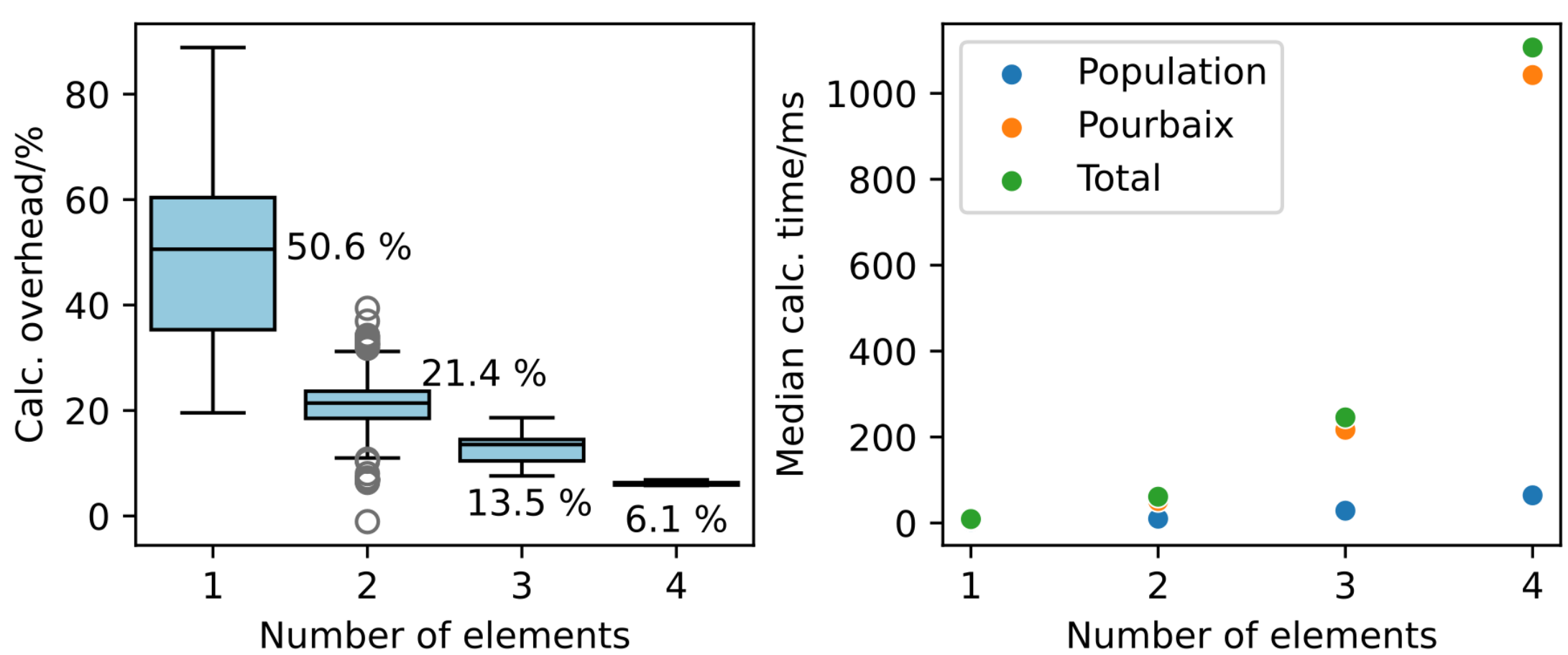

Figure S2: Median timings across 39 phases and 10 replicas. Left, percentual overhead of population against direct Pourbaix diagram calculation, respect to the number of elements entering the diagram. Right, actual computation times in ms for population calculation, Pourbaix diagram generation, and total time.

From Figure S2, we have that while the overhead is relatively large (around 50 %) for the most simple mono-elemental Pourbaix diagrams, it quickly decreases for binary, ternary or more complex combinations. Moreover, from the right panel, we observe how as the complexity of the system under study increases, the bottleneck of the calculation is always the diagram calculation (which has to deal

with the complex combinatorics of the system), confirming the unexpensiveness of the population estimation.

4 Speciation of metal alloys

For reference, we also constructed population vs. pH plots fixing V = 0.0 V for the metal alloys discussed in the main text.

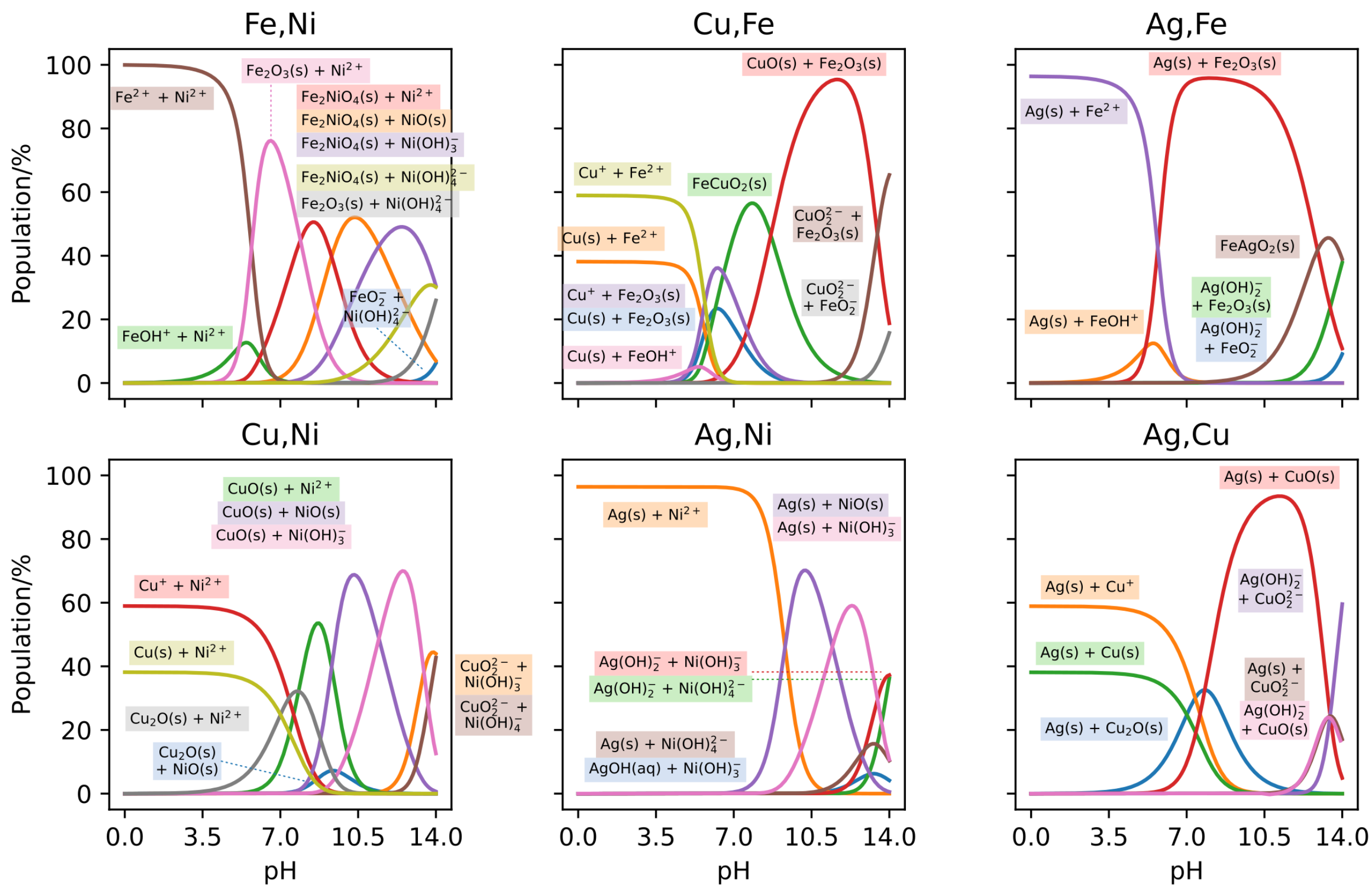

Figure S3: Speciation diagrams for A/B binary metal combinations: 50% A, 50% B, pH range 0 to 14, potential V = 0.0 V. Color scale is qualitative.

5 Speciation of Bi/Sb/Te mixtures

Apart from the neutral pH situation presented in the main text, we also explored the distribution of Bi/Sb/Te phases at V = 0.0 V for acidic (0.5) and alkaline (13.5) pHs with varying proportions of bismuth and antimonium (Figures S4 and S5).

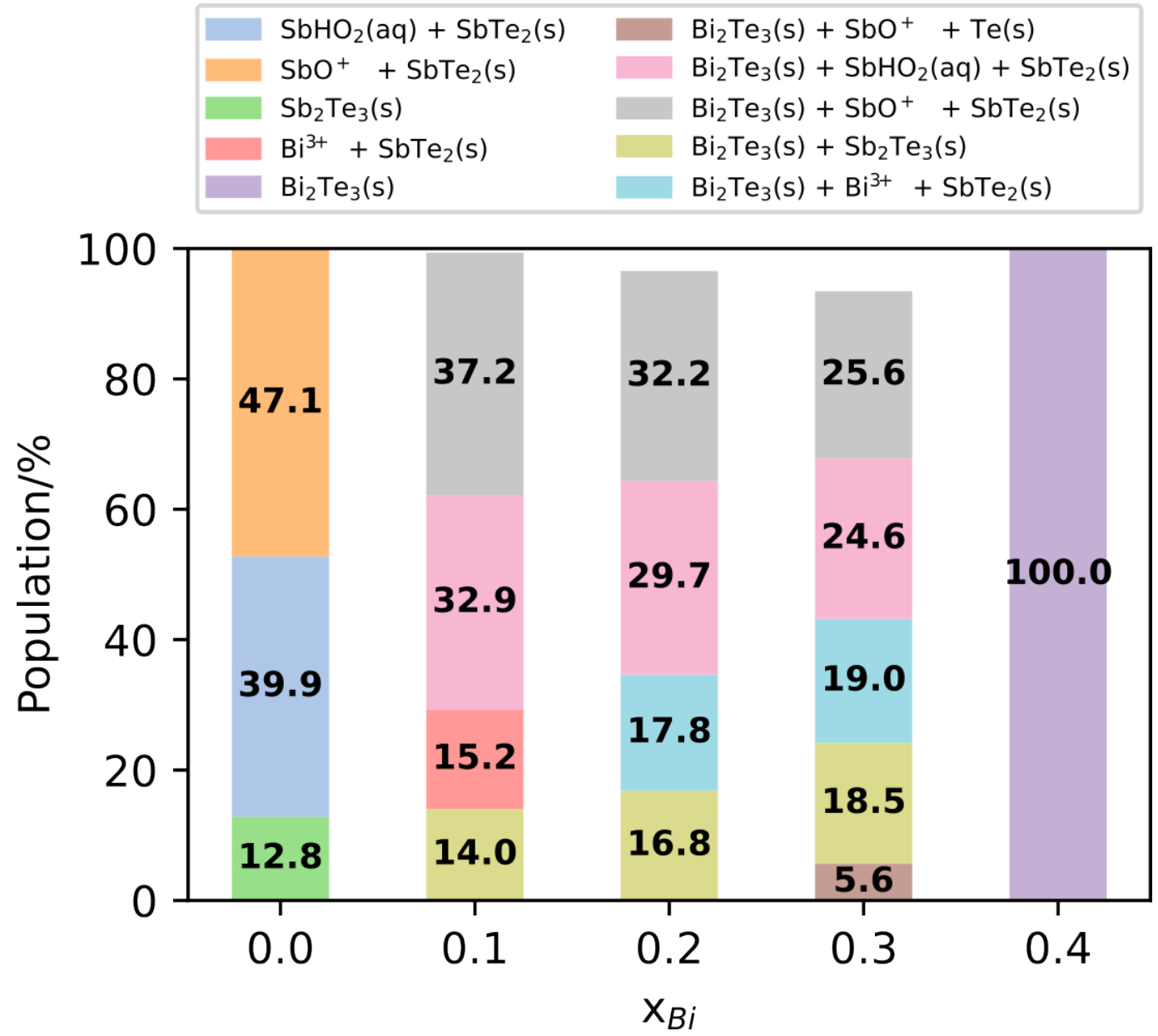

Figure S4: Main speciation of Bi/Sb/Te system at pH = 0.5 and V = 0.0 V, with varying amounts of Bi (0.0 to 0.4). Only the 15 major phases and these with populations above 5.0 % are included, leading to total population being lower than 100%.

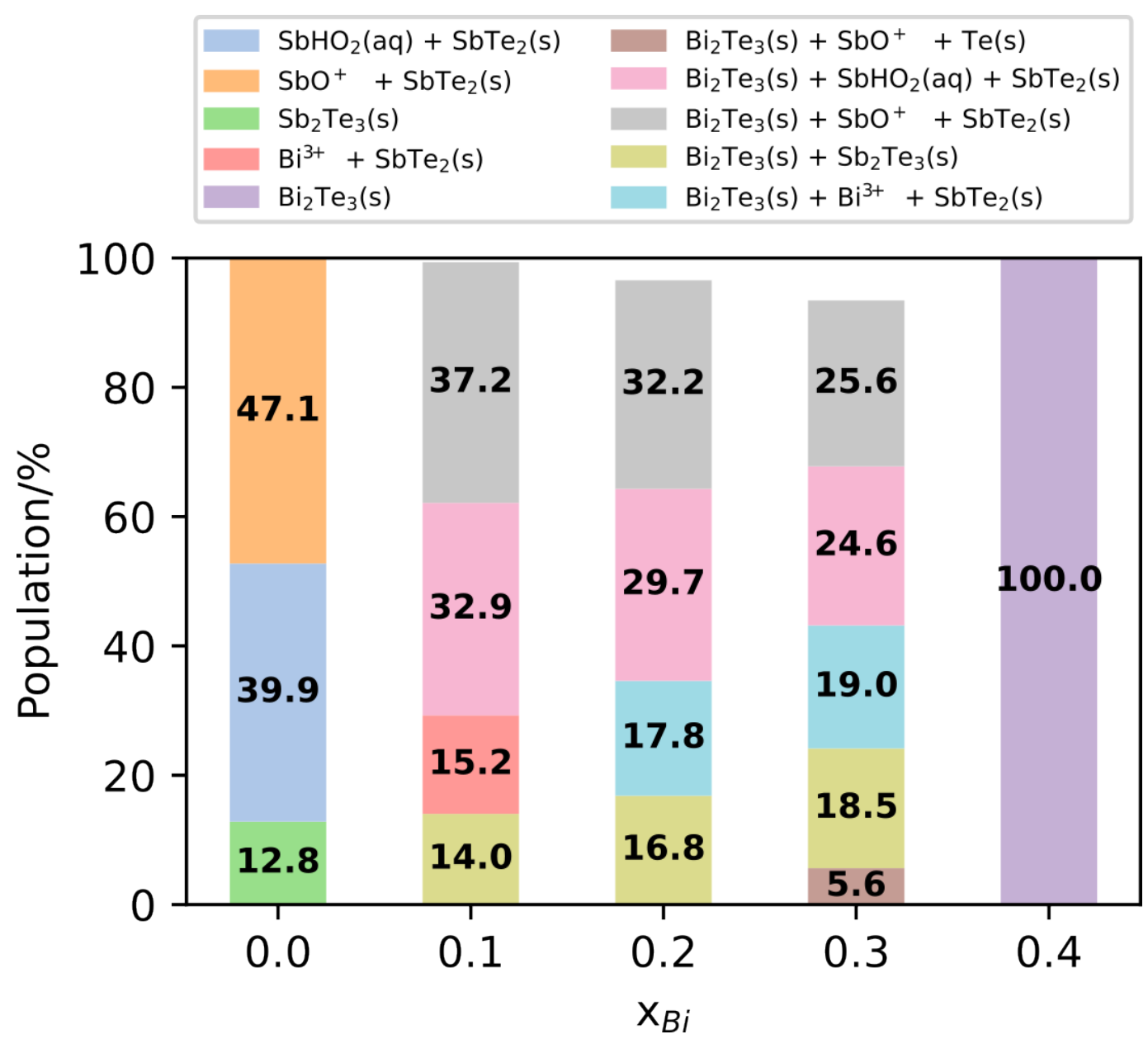

Figure S5: Main speciation of Bi/Sb/Te system at pH = 13.5 and V = 0.0 V, with varying amounts of Bi (0.0 to 0.4). Only the 15 major phases and these with populations above 5.0 % are included, leading to total population being lower than 100%.

A brief discussion of Figures S4 and S5 is provided in the main text.

6 Grid sensitivity

Across the main text, we have used a default pH/potential grid of 150x150 (22500 points) for the evaluation of phase populations. However, this is a somehow arbitrary choice, as grid size is an adjustable parameter: while 150x150 provided reasonable results, with population-based limits aligning perfectly with traditional Pourbaix lines, we can also explore the effect of smaller and larger grids in the obtained Pourbaix spaces.

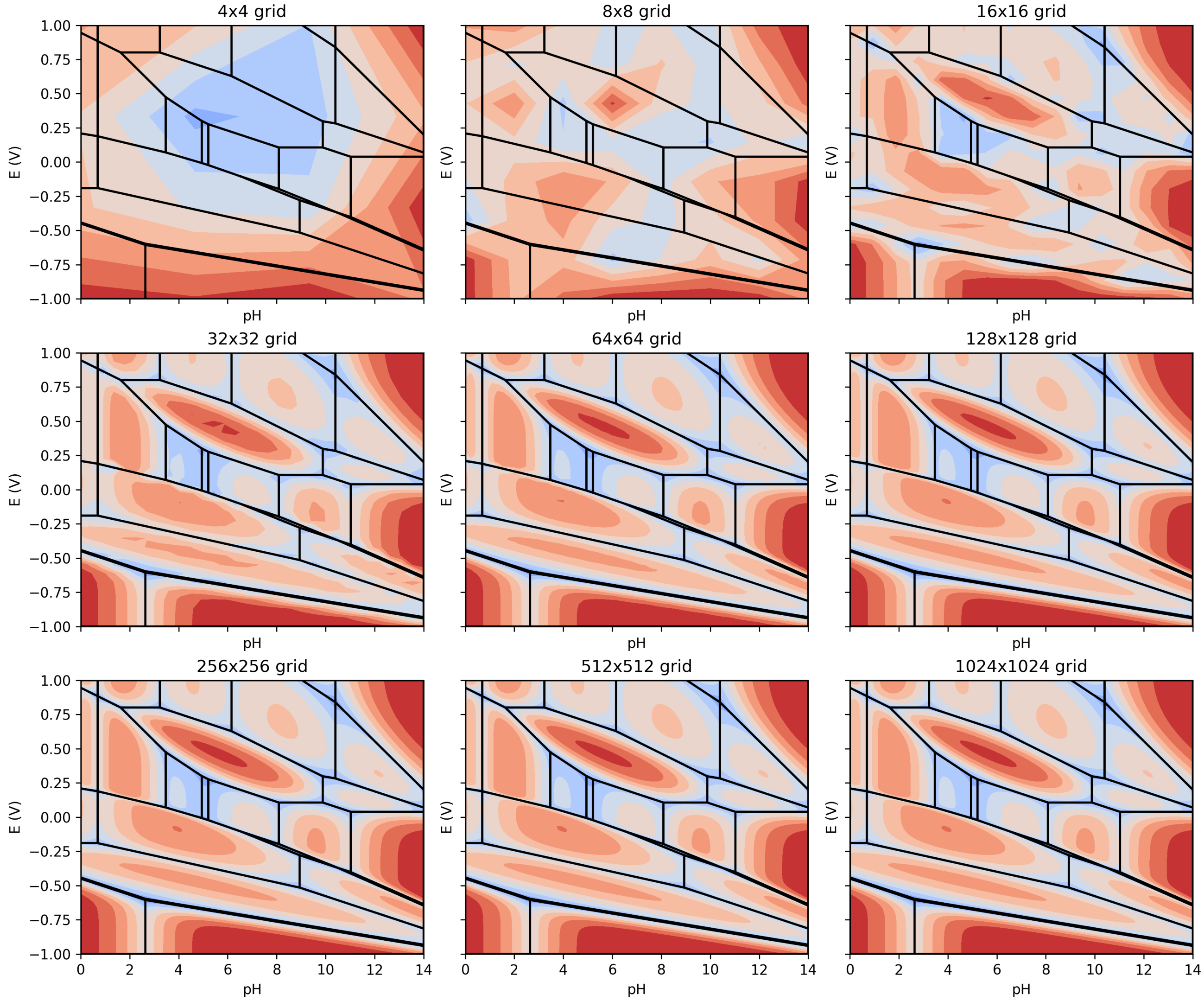

Figure S6: Boltzmann-weighted Pourbaix diagram for Bi/Te system: 50% Bi, 50% Te, V range (vs. SHE) -1.0 V to 1.0 V, pH range 0 to 14, for varying grid sizes (indicated in the plot title). Color scale corresponds to the population of the major phase, labeled in the graph.

Figure S6 illustrates the alignment of traditional Pourbaix limits with the ”soft” limits defined by phase populations. The two smallest grids (4x4 and 8x8), where only 16 and 64 population values are evaluated do clearly fail in delimiting the different regions, due to lack of resolution: the changes in both pH and potential between consecutive points in the grid are way too large for transitions having any smoothness — or, in other words, the sampling is insufficient. From there, 16x16 (256 points) starts to delimit some of the regions, particularly at the lower part of the map (highly reductive potentials), and 32x32 (1024 points) already shows a reasonable outline of the phase distribution. However, is from 64x64 (4096 points) and further on where the population contours become fully smooth. Differences between 128x128, 256x256, 512x512 and 1024x1024 are minimal and practically imperceptible. Consequently, the 150x150 grid used across the text is confirmed as a reasonable choice, providing a good resolution while keeping the number of total population evaluations moderate. Nonetheless, this guideline may vary depending on the system (as well as on the desired pH and/or potential ranges), and for complex situations it would be advisable to do initial grid size tests to ensure adequacy and/or avoid increasing the computational cost.

7 Application to stainless steel alloys

As a more specific example, we considered stainless steels, mainly composed by iron and chromium — although other elements (like carbon, nickel, or molybdenum) are often also present for industrially employed alloys. In this context, the study of possible corrosion pathways, particularly in aggressive conditions (strong acidity or alkalinity, highly oxidizing or reductive potentials, large temperatures...) is of the uttermost importance to characterize the applicability ranges of the materials. Pourbaix diagrams can provide a broad view of the electrochemical windows for corrosion, immunity and passivation on these materials, thus being a valuable exploratory tool. However, we should also recall that kinetic effects, which are also essential for a full understanding of corrosion, cannot be considered under Pourbaix-based frameworks that are purely thermodynamic.

Although a thorough study of stainless steels is out of the scope of this communication, due to the complexity of these materials, we considered an initial model study to further contextualize the relevance of our methodology. In this context, we will consider a 80% Fe/20 % Cr alloy (in line with the Cr content of common stainless steels such as 204 or 304), which we will compare with the studies from Malki et al.[2] and Muñoz-Portero et al.[3], who recently targetted the applicability of Pourbaix diagrams to Fe/Cr alloys.

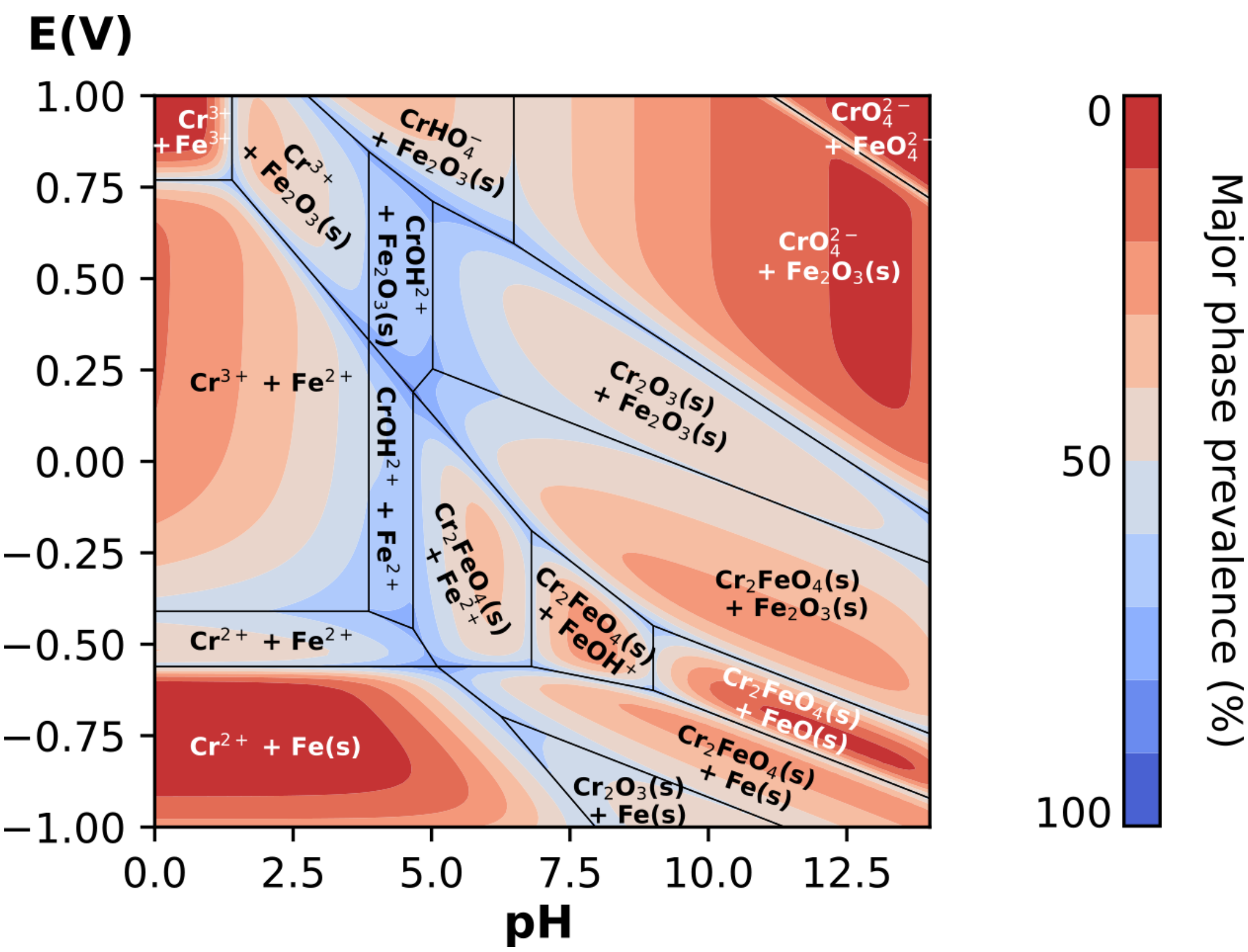

Figure S7: Boltzmann-weighted Pourbaix diagram for Fe/Cr system: 80% Fe, 20% Cr, V range (vs. SHE) -1.0 V to 1.0 V, pH range 0 to 14. Color scale corresponds to the population of th major phase, labeled in the graph.

A relevant feature of the diagram is the transition between dissolved ions and the solid Cr_2FeO_4(s) phase, marking a pathway for the loss of the passivating chromium/iron oxide layer on a potential window between -0.5 and 0.5 V. Muñoz-Portero and co-workers[3] report the displacement of the phase transition between Fe^{2+} and $FeCr_2O_4$ depending on ion activity, which here is fixed to $1.0 \cdot 10^{-6}$M, following Materials Project standards — however, this parameter can be tweaked via PourbaixUP if desired. When this activity is larger, this transition line moves to more acidic pH values, enlarging the passivation region. Looking at this region in our competition-informed diagram (Figure S7),

we encounter a wide zone with low maximum prevalences (below 50%), with a strong competition between Cr^{3+}, $Cr(OH)^{2+}$ and Cr_2FeO_4. This competition region aligns well with the expected changes in the passivation window, and depicts a smoother switch between passivation and corrosion zones in comparison with the hard transitions associated to traditional diagrams.

On the other hand, Malki et al.[2] also consider the free energies from the Materials Project, applying systematic perturbations to characterize metastability regions. One of the main features explored in that study is the extension of the domain of the $Fe + Cr_2O_3$ phase at alkaline pH and reductive potentials upon its free energy stabilization, overtaking Cr_2FeO_4. Looking at this region in Figure S7, we find competition between both oxides (pH between 8 - 12 and potentials between -0.75 and -1.00 V), which again aligns well with the observations based on free energy perturbation. Cr_2O_3 should not be disregarded when considering the behavior of this system in alkaline media and reductive potentials, despite seeming absent from the corresponding Pourbaix region.

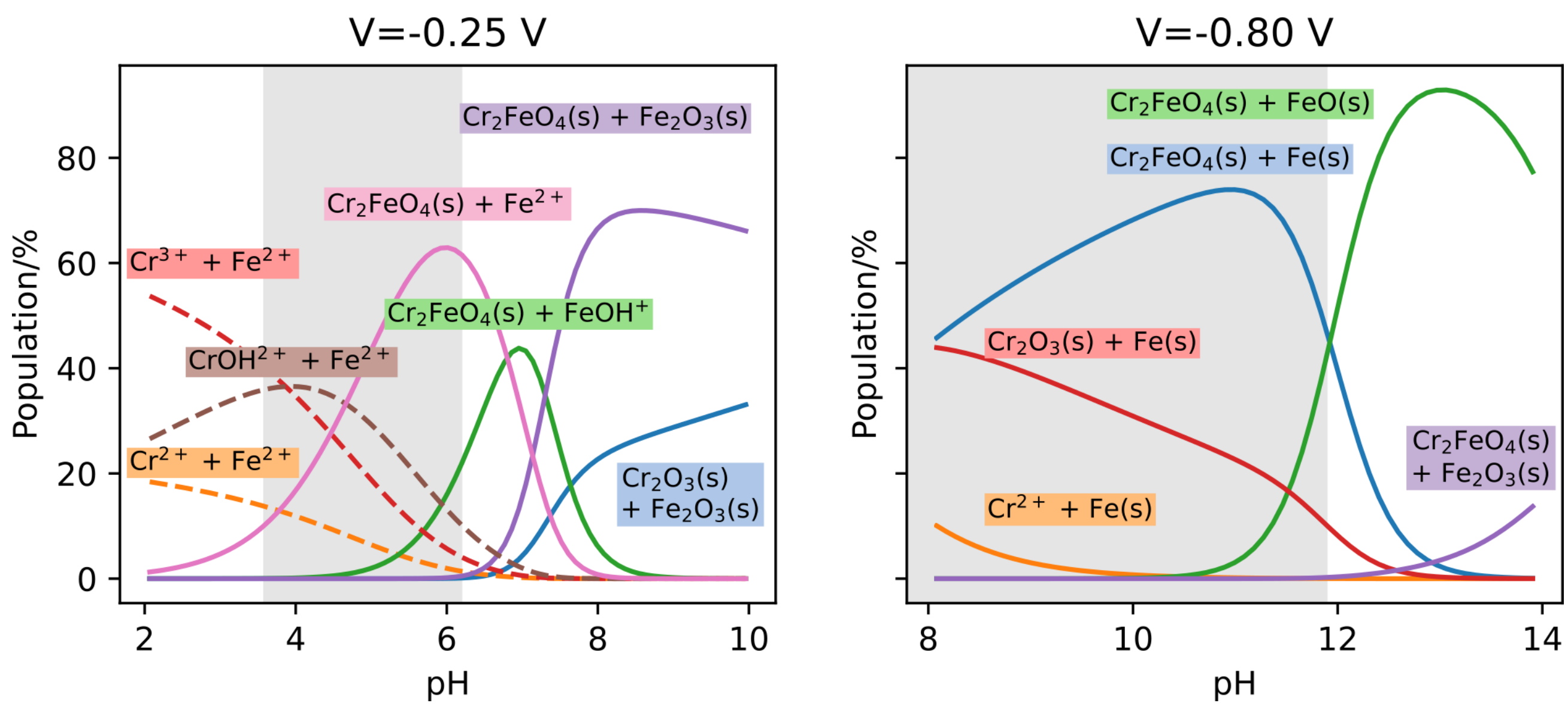

Figure S8: Population vs. pH speciation diagrams for Fe/Cr system: 80% Fe, 20% Cr in relevant regions of the Pourbaix space. Left, V = -0.25 V and pH between 2 and 12 (Cr_2FeO_4 corrosion). Right, V = -0.8 V and pH between 8 and 14 (Cr_2FeO_4 and Cr_2O_3 competition). Color scale is qualitative and independent between the panels. Grey shading highlights regions where the minor phase in competition is above 10% population.

Figure S8 confirms the trends shown in the competition-informed Pourbaix diagram at fixed potential values, selected to cover the main transition regions for the aforementioned processes: acidic corrosion of Cr_2FeO_4 in Fig. S8A and alkaline interplay between Cr_2FeO_4 and Cr_2O_3 in Fig. S8B.

References

[1] W. Sun, D. A. Kitchaev, D. Kramer, and G. Ceder, "Non-equilibrium crystallization pathways of manganese oxides in aqueous solution," *Nature Communications*, vol. 10, p. 573, Feb. 2019.

[2] B. Malki, I. Guillotte, and B. Baroux, "Deriving Metastable Pourbaix Diagrams of Stainless Steels Using Density Functional Theory Calculations," *Journal of The Electrochemical Society*, vol. 170, p. 091501, Sept. 2023.

[3] M. Muñoz-Portero, T. Nachiondo, and J. García-Antón, "Pourbaix diagrams for iron-chromium alloys in lithium bromide absorption machines," *Electrochimica Acta*, vol. 497, p. 144545, Sept. 2024.
